# A Feature-Based Approach to Modeling Protein–DNA Interactions

**DOI:** 10.1371/journal.pcbi.1000154

**Published:** 2008-08-22

**Authors:** Eilon Sharon, Shai Lubliner, Eran Segal

**Affiliations:** 1 Department of Computer Science and Applied Mathematics, Weizmann Institute of Science, Rehovot, Israel; 2 Department of Molecular Cell Biology, Weizmann Institute of Science, Rehovot, Israel; Washington University, United States of America

## Abstract

Transcription factor (TF) binding to its DNA target site is a fundamental regulatory interaction. The most common model used to represent TF binding specificities is a *position specific scoring matrix* (PSSM), which assumes independence between binding positions. However, in many cases, this simplifying assumption does not hold. Here, we present *feature motif models* (FMMs), a novel probabilistic method for modeling TF–DNA interactions, based on *log-linear models*. Our approach uses sequence *features* to represent TF binding specificities, where each feature may span multiple positions. We develop the mathematical formulation of our model and devise an algorithm for learning its structural features from binding site data. We also developed a discriminative motif finder, which discovers de novo FMMs that are enriched in target sets of sequences compared to background sets. We evaluate our approach on synthetic data and on the widely used TF chromatin immunoprecipitation (ChIP) dataset of Harbison et al. We then apply our algorithm to high-throughput TF ChIP data from mouse and human, reveal sequence features that are present in the binding specificities of mouse and human TFs, and show that FMMs explain TF binding significantly better than PSSMs. Our FMM learning and motif finder software are available at http://genie.weizmann.ac.il/.

## Author Summary

Transcription factor (TF) protein binding to its DNA target sequences is a fundamental physical interaction underlying gene regulation. Characterizing the binding specificities of TFs is essential for deducing which genes are regulated by which TFs. Recently, several high-throughput methods that measure sequences enriched for TF targets genomewide were developed. Since TFs recognize relatively short sequences, much effort has been directed at developing computational methods that identify enriched subsequences (motifs) from these sequences. However, little effort has been directed towards improving the representation of motifs. Practically, available motif finding software use the position specific scoring matrix (PSSM) model, which assumes independence between different motif positions. We present an alternative, richer model, called the feature motif model (FMM), that enables the representation of a variety of sequence features and captures dependencies that exist between binding site positions. We show how FMMs explain TF binding data better than PSSMs on both synthetic and real data. We also present a motif finder algorithm that learns FMM motifs from unaligned promoter sequences and show how de novo FMMs, learned from binding data of the human TFs c-Myc and CTCF, reveal intriguing insights about their binding specificities.

## Introduction

Precise control of gene expression lies at the heart of nearly all biological processes. An important layer in such control is the regulation of transcription. This regulation is preformed by a network of interactions between transcription factor proteins (TFs) and the DNA of the genes they regulate. To understand the workings of this network, it is thus crucial to understand the most basic interaction between a TF and its target site on the DNA. Indeed, much effort has been devoted to detecting the TF–DNA binding location and specificities.

Experimentally, much of the binding specificity information has been determined using traditional methodologies such as footprinting, gel-shift analysis, Southwestern blotting, or reporter constructs. Recently, a number of high-throughput technologies for identifying TF binding specificities have been developed. These methods can be classified into two major classes, in vitro and in vivo methods. In vitro methods can further be classified to methods that select high-affinity binding sequences for a protein of interest [1],[2] (reviewed in Elnitski et al. [3]), and high-throughput methods that measure the affinities of specific proteins to multiple DNA sequences. Examples of the latter class of methods include protein binding microarrays [4]–[6] and microfluidic platforms [7], which claim to achieve better measurement of transient low affinity interactions. The in vivo methods are mainly based on microarray readout or high throughput sequencing technologies readout of either DNA adenine methyltransferase fusion proteins (DamID) or of chromatin immunoprecipitation DNA-bound proteins (ChIP-chip, ChIP-PET, ChipSeq/Chip-seq) [8]–[16]. The in vivo methods were recently used to characterize the binding specificities of all TFs in the yeast *Saccharomyces cerevisiae* [8], [9], [17]–[21] and, more recently, to identify genomic targets in mammalian cells [10]–[16], [22]–[26].

However, despite these technological advances, distilling the TF binding specificity from these assays remains a great challenge, since in many cases the in vivo measured targets of a TF do not have common binding sites, and in other cases genes that have the known and experimentally determined site for a TF are not measured as its targets. For these reasons, the problem of identifying transcription factor binding sites (TFBSs) has also been the subject of much computational work (reviewed by Elnitski [3]). The most common approaches start by defining sets of genes that are potentially coregulated, either from clusters of coexpressed genes in microarray data [27], from functional annotations of genes [28], or from TF chromatin immunoprecipitation [17],[29]. They then attempt to identify regulatory elements by searching for common motifs in the promoter regions of the genes in each group [30]–[35]. Recently, Eden et al. [36] developed a discriminative motif finder that is well suited for finding motifs in a ChIP-chip experiment. Their method combines the search for a cutoff that defines the positive set, with the search for a motif that discriminates the positive set from the rest of the chip probes. Other approaches work in the opposite direction, by first reducing the sequence data into some predefined features of the gene (e.g., presence or absence of all DNA-words of length 6–7), and then identifying the putative binding sites, for example, by keeping the features, or combinations thereof, whose genes are coexpressed [37]–[39]. Recently, this latter approach was extended to comparative genomic methods that filter the initial library of features to those that show high conservation in evolutionarily closely related species [40]–[42].

The experimental and computational approaches above revealed that TFBSs are short, typically 6–20 base pairs, and that some degree of variability in the TFBSs is allowed. For these reasons, the binding site specificities of TFs are described by a sequence *motif*, which should represent the set of multiple allowed TFBSs for a given TF. The most common representation for sequence motifs is the *position specific scoring matrix* (PSSM), which specifies a separate probability distribution over nucleotides at each position of the TFBS. The goal of computational approaches is then to identify the PSSM associated with each TF.

**Figure 1:**
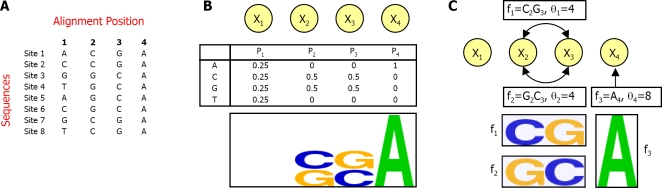
Comparison between FMMs and PSSMs in a toy example of a TFBS with four positions. (A) Eight input TFBSs that the TF recognizes. (B) A PSSM for the input data in (A), showing its log-linear model network representation, probability distributions over each position, and sequence logo. Note that the PSSM assigns a high probability to CG and GC in positions 2 and 3 as expected by the input data, but it also undesirably (and unavoidably) assigns the same high probability to CC and GG in these positions. (C) An FMM for the input data in (A), showing the associated log-linear model network, with 3 features and sequence logo. Note that features *f*_1_ and *f*_2_ assign a high probability to CG and GC in positions 2 and 3 but not to CC and GG in these positions, as desired.

Despite its successes, the PSSM representation makes the strong assumption that the binding specificities of TFs are position-independent. That is, the PSSM assumes that for any given TF and TFBS, the contribution of a nucleotide at one position of the site to the overall binding affinity of the TF to the site does not depend on the nucleotides that appear in other positions of the site. In theory, it is easy to see where this assumption fails. For example, consider the models described in Figure 1. The TFBS (Figure 1A) data contains only “CG” or “GC” in the center positions. Although the PSSM learned from this data (Figure 1B) assigns high probability to these nucleotide pairs, it also undesirably (and unavoidably) assigns high probability to “CC” and “GG” in the center positions. However, if instead of the PSSM representation, we allow ourselves to assign probabilities to multiple nucleotides at multiple positions, we could use the same number of parameters to specify the desired TF binding specificities (For example, consider the model illustrated in Figure 1C, which uses two parameters that are each associated with two positions to give exact description of the binding specificities over the center positions). This observation lies at the heart of our approach.

From the above discussion, it should be clear that the position-independent assumption of PSSMs is rather strong, and that relaxing this assumption may lead to a qualitatively better characterization of TF motifs. Indeed, recent studies revealed specific cases in which dependencies between positions may exist [7],[43]. Several models were developed to capture such dependencies (see [44] for a brief review). These models can be classified into two main classes: Markov chains based models [44],[45] and Bayesian Network based models [46]–[49]. In the first class, the dependencies between neighboring positions are modeled using a Markov model of some order. A recent representative of this class is the permutated variable length Markov model (PVLMM) of Zhao et al., which incorporates two major improvements: it searches for the best permutation of the motif positions, and it reduces the number of parameters by using a context tree representation for the Markov model representation. Although Markov chain based models may perform well in some datasets, they have a limited ability to model dependencies between more distant positions. Since modeling these dependencies by increasing the order of the Markov model exponentially increases the size of the model representation, it was suggested to search for a permutation of the binding site positions that produces the best model. However, this is only a partial solution, as it poses a limitation on the model learned and easily becomes computational intensive for long motifs (Zhao et al. limit their motifs to length 9 bp).

The second class of models was proposed by Barash et al. [46], who developed a Bayesian network approach to represent higher order dependencies between motif positions. They showed that these models predict putative TFBSs in ChIP-chip data with higher accuracy than PSSMs. Ben-Gal et al. [47] extended this approach by using a context dependent representation of the conditional probability distributions, which, to some extent, reduces the representation size (depending on the data). Zhou et al. suggested a simpler Bayesian network model (GWM) where only dependencies between nonoverlapping positions are modeled [49]. Pudimat et al. also extended the Bayesian network framework by adding structural DNA features [48]. However, the Bayesian network representation, due to its acyclicity constraints, imposes nonnatural restrictions on the motif structure, and its conditional probability distributions limit the number of dependencies that can be introduced between positions in practice, due to the exponential increase in the number of parameters introduced with each additional dependency. Although some of these issues may be addressed, e.g., using sparse conditional probability distribution representations, Bayesian networks do not seem to be the ideal and most intuitive tool for the task.

Another class of TF binding specificities models that is complementary to the above two is a mixture of models. In the above mentioned work, Barash et al. also used a mixture of PSSMs to model TFBSs. In this representation, each motif is modeled as a mixture of PSSMs each defining a different mode of binding. This approach was later extended as a part of the LOGOS [50] and MotifBooster [51] motif finding software. However, this approach does not explicitly represent dependencies between binding site positions.

Here, we propose a novel approach for modeling TFBS motifs, termed *feature motif models* (FMMs). Our approach is based on describing the set of sequence properties, or *features*, that are relevant to the TF–DNA interactions. Intuitively, the binding affinity of a given site to the TF increases as it contains more of the features that are important for the TF in recognizing its target site. In our framework, features may be binary (e.g., “C at position 2, and G at position 3”) or multivalued (e.g., “the number of G or C nucleotides at positions 1–4”), and global features are also allowed (e.g., “the sequence is palindromic”). Each feature is assigned a statistical weight, representing the degree of its importance to the TF–DNA interaction, and the overall strength of a TFBS can then be computed by summing the contribution of all of its constituent features. We argue that this formulation captures the essence of the TF–DNA interaction more explicitly than PSSMs and other previous approaches. It is easy to see that PSSMs are a special case of FMMs, since a PSSM can be described within our framework using four single nucleotide features per position. Our approach can also naturally represent complex and distant dependencies efficiently, thereby overcoming a limitation of other models that have been proposed.

The rest of the paper is organized as follows: The Results section starts with a brief overview of our methodology. We then validate our approach by learning FMMs from synthetic and real aligned TFBS data. Next, we devise a novel motif finder algorithm that finds motifs in a set of unaligned sequences, and validate its performance on yeast TF ChIP data [17]. Using this motif finder, we demonstrate the benefits of using FMMs instead of PSSMs as a basic building block of a motif finder, which represents TF binding motifs. Finally, we present insights that we gained from learning FMMs for two human TFs, CTCF and c-MYC. In the Methods section we discuss the details and the mathematical formulation of our FMM approach. The problem of learning an FMM from TFBS data is quite difficult, as it reduces to structure learning in Markov networks, a paradigm that is still poorly developed. In the Methods section we elaborate on our learning strategy and suggest an improved methodology for optimizing the data likelihood, which we define as our objective function.

## Results

### Framework and Algorithms in a Nutshell

We first briefly describe the FMM representation, and how it is learned from aligned TFBS sequences. Next, we give a high-level view of our motif finder, that finds motifs in unaligned sequences, and allows their representation as FMMs. All of the algorithms described here are available as downloadable software or as an online web service at our web site: http://genie.weizmann.ac.il/. See the Methods section for a more elaborate description.

#### Feature motif model (FMM)

As mentioned above, we represent TF binding specificities as the set of *sequence features* (denoted by *f*), which contribute to the binding interaction. Although our framework can handle various definitions of *sequence features*, in this work we focus on features that are indicators for the appearance of specific nucleotides in a specific set of one or two positions (as in the above example: “C at position 2, and G at position 3”). It is easy to see that our model can represent PSSMs, by defining the set of all possible single position features (of the type: “A at position 1”). However, it can also account for dependencies between different positions of the TF binding motif by defining features that span two positions. A representation of Markov networks, which is often referred to as log-linear models [52], is a natural framework for compact representation of a distribution as a set of feature functions. Intuitively, in this framework, each feature *f_k_* is associated with a weight *θ_k_*, representing its contribution to the binding affinity. Given a sequence x, we compute its binding probability by summing over all the weights of the features that appear in the sequence:
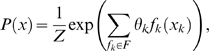
where *Z* is a normalization factor and *x_k_*are the domain positions of *f_k_*. A motif is then defined as a set of features and a set of weights associated with these features. Given a binding position *x_i_*, its Markov blanket 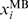 is defined as all the other positions that are in some feature that contains *x_i_*. Thus, the motif model encodes the following independence assumptions: each positon is independent of all other positions given the sequence of its Markov blanket: 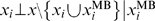. This definition does not pose any limitations on the dependencies that may be learned. The log-linear representation is equivalent to any other representation of a Markov network. In terms of Markov networks, our motif can be represented as an undirected graph with a vertex for every binding position and a clique over each feature domain (see Figure 1 for an example). The parameters of the network are defined by the exponent of the feature weights. Thus, learning the set of features is equivalent to learning a Markov network structure and learning the features weights is equivalent to learning the network parameters. We will therefore use the term log-linear model to describe a log-linear representation of Markov networks. Given a dataset *D* = {*x*[1],…,*x*[*N*]} of *N* aligned i.i.d TFBSs, our aim is to optimize the data likelihood over all possible models. We hence define our objective function *F* as the data log likelihood 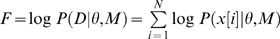, where *M* denotes the model and *θ* denotes the sets of weights.

A detailed description of the learning process is presented in the Methods section. Here we briefly describe the general flow of learning an FMM from TFBS data. First, for every possible feature of maximum domain size *D* (here *D* = 2), we count its number of appearances in the data. For example, we count the number of times that the feature “G in position 2 and A in position 4” appears in the data. There are at most *O*(*L^D^*) such possible features (where *L* is the motif length), but a much smaller number of features typically exists in TFBSs data (see Protocol S1 for more details). Next, we reduce the feature space using a Binomial test to evaluate the statistical significance of features that span more than one position (as described in the Methods section). The test evaluates the statistical significance of observing the number of feature appearances, given the single position nucleotides empirical distributions (as evaluated by the single position appearances counts). We filter out nonsignificant features using FDR [53] (using a threshold of 0.2). We then use the *grafting* methodology of Perkins et al. [54], which optimally selects features from the feature space according to their gradient in the objective function. In order to control for model complexity and to achieve a sparse representation, we use the *L*_1_-Regularization suggested by Lee et al. [55], which penalizes models linearly by their sum of weights. We therefore modify our above objective function *F* by adding to it a linear penalty term, resulting in
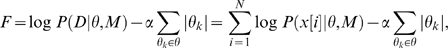
where *α* is a free parameter of the *L*_1_ penalty term of our objective function. This process of features selection is guaranteed to converge. Finally, the output FMM is represented using a simple sequence logo as in the example given in Figure 1. In this logo, each indicator function feature is represented by a box. The horizontal position of the box and the nucleotides that are written inside it, define the indicator function. The height of the box is linear with respect to the expectation of the feature according to the model (as computed in Equation 4 in the Methods section). Features over more than one position have a gray background. The problem of representing complex dependencies in a relatively simple and readable logo is not trivial. Nevertheless, a clear logo is important for easy interpretation of the results. On our web site, we also offer different logo representations and an XML format representation of the model. Our model logo is very useful for deriving hypotheses on specific TF binding specificities, and on dependencies between the motif positions.

#### FMM motif finder

**Figure 2:**
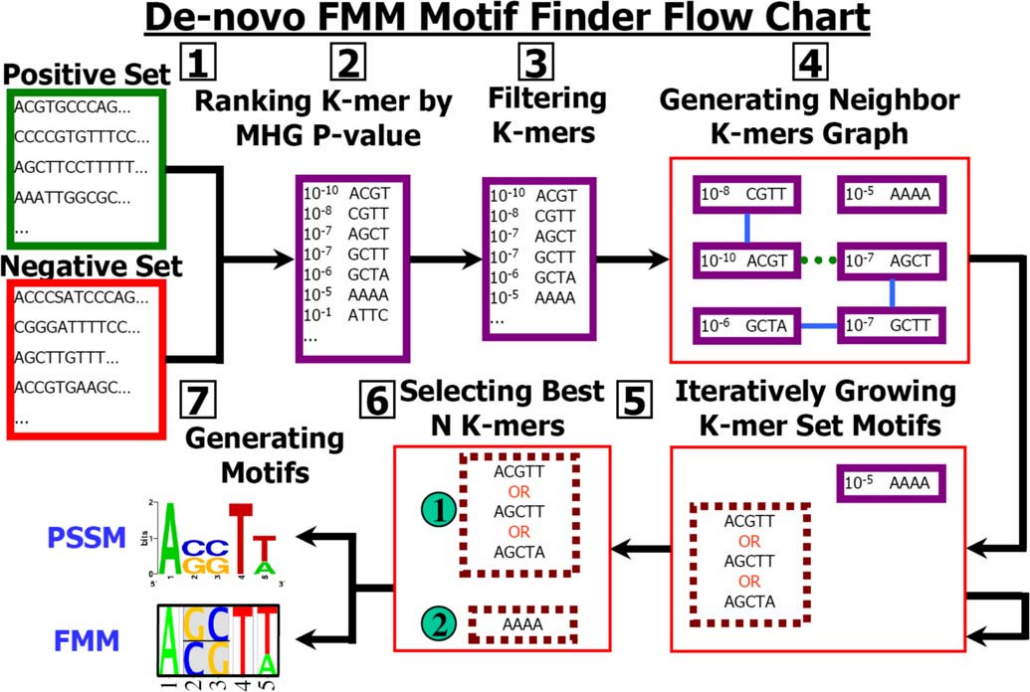
FMM motif finder flow chart. The algorithm gets as input (1) sets of positive and negative (in terms of TF binding) unaligned sequences. It then (2) computes for every possible *K*-mer its enrichment multidimensional hypergeometric *p*-value (MHG *p*-value) by finding all its occurrences in the input sequences. The *K*-mers are ranked by the MHG *p*-value. (3) Non-significant K-mers are then filtered, using FDR controlled threshold. Next (4) an undirected graph is built. Each *K*-mer is a vertex, and two *K*-mers are connected with an edge if their Hamming distance is smaller then *H*_Distance_ or if they can be aligned without mismatches with a relative shift of up to *M*_Shift_ (here blue line edge stands for Hamming distance 1 and dotted green edge for Hamming distance 2). The algorithm then (5) iteratively selects the most significant *K*-mer in the graph and grows a KMM along the edges as described in the text. After each *K*-mer is associated with exactly one KMM set, (6) all the sets are ranked according to their MHG *p*-value, and the best *M* sets are chosen. The process is repeated for every *K*_min_≤*K*≤*K*_max_ and again the overall *M* best KMM are chosen. Each of the chosen KMMs is used (7) to produce either an FMM or a PSSM motif in the method described in the text. As a last step similar motifs are removed.

As a proof of concept, we developed a novel motif finder software and used it to compare the FMM to the PSSM as models for motif representation, within a de novo motif finding process. Our motif finder follows a discriminative methodology, which means that it finds motifs that are enriched in a positive set of unaligned sequences compared to a negative set of unaligned sequences. It receives as input a set of unaligned sequences that a TF binds to (positive set), and a background set of unaligned sequences that are not bound by the TF (negative set). The motif finding scheme consists of two main steps: In the first, we extract all sequences of length *K* (referred to as “*K*-mers”) and greedily grow motifs (defined by a set of OR and AND operations on a set of *K*-mers) that are discriminatively enriched in the positive set over the negative set. We refer to such motifs as *K*-mer set motif models, or “KMM”s. An important property of KMMs is that they preserve dependencies between motif positions, unlike most of the commonly used motif representations (e.g., PSSMs, Hamming balls, etc.). This property is the essence of how our motif finder can later produce FMM motifs that accurately represent the data. Another advantage of the KMM methodology is that KMMs maintain an alignment of their *K*-mers, which induces the motif length (see Protocol S1 and Figure S1). Thus, our algorithm does not require the motif length as input. The enrichment measure we use is the multidimensional hyper-geometric *p*-value (MHG *p*-value), suggested by Eden et al. [36], as described in the Methods section (Finding De Novo FMM Motifs). This measure takes into account the ratio between the number of motif hits in the positive set and the number of motif hits in the negative set. The higher the ratio, the smaller the MHG *p*-value, indicating higher enrichment. In the second step, each enriched KMM is used for extracting aligned TFBSs from the positive set, from which a motif model, FMM or PSSM, is learned. The scheme is illustrated in Figure 2 and described in details in the Methods section (Finding De Novo FMM Motifs). We used this two step scheme in order to generate high quality data for an FMM-PSSM comparison.

### Results Overview

We now present an experimental evaluation of our FMM learning approach. First, we used synthetic data to tune the free parameter of the penalty term and to test whether our method can reconstruct sequence features that span multiple positions when these are present. We then compared the ability of our approach to that of PSSMs on learning real binding site specificities of human TFs from two datasets of TFBS [10],[13]. Next, we validated the ability of our motif finder to find TFBS motifs in yeast ChIP-microarry data [17]. We show that its performance is comparable to state-of-the-art motif finders. Finally, we compiled a collection of high throughput human and mouse TF ChIP datasets, and used our motif finder to learn de novo motifs for each of the TFs. We show that our FMM approach learns the binding specificities of these TFs better than the PSSM approach.

### FMMs Reconstruct Binding Specificities from Synthetic Aligned TFBS Data

**Figure 3:**
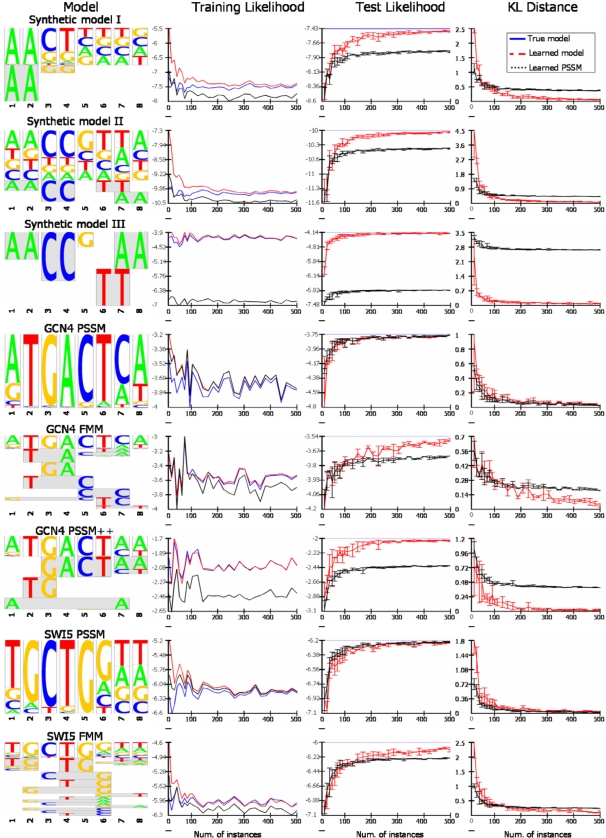
Evaluation of our approach on synthetic data. Results are shown for eight manually constructed models, from which we drew samples and constructed FMMs and PSSMs. The presented models from top down are three synthetic models. A PSSM and an FMM learned from MacIsaac et al. [29] data for the TF GCN4. GCN4 MacIsaac et al. A PSSM learned from the MacIsaac et al. [29] data for the GCN4 transcription factor, with manually addition of eight synthetic features over two positions each (referred as GCN4 PSSM++). A PSSM and an FMM learned from MacIsaac et al. data for the TF SWI5. For each model, shown are its sequence logo (left), training and test log-likelihood (average per instance for the true model, and learned FMM and PSSM) and KL distance of the learned FMM and PSSM models from the true model (train likelihood error bars were excluded for clarity). The height of each feature in the sequence logo is linear with respect to its expectation. Features over more than one position have gray background.

Before integrating our algorithm for learning FMM from aligned TFBS data into our motif finder algorithm, we separately evaluated it in a controlled setting. As an initial test for our method, we wanted to evaluate the ability of our algorithm to learn sequence features that span multiple positions when such exist, and to avoid learning such features when none exist. For this purpose, we manually created eight sequence models of varying weights and features (which we will refer to as “true” models), and learned both PSSM and FMMs from aligned TFBSs that we sampled from them (Figure 3). Our eight sequence models contained three manually-curated models with features over single and double positions (we denoted these models as Synthetic model I–III). In order to make sure that our model does not learn double position features when none exist, we used as our true model the PSSMs of yeast GCN4 and SWI5 TFs from MacIsaac et al. [29] (denoted GCN4 PSSM and SWI5 PSSM). Even though the relatively small size of the MacIsaac et al. dataset may cause overfitting when used directly for FMM learning, sampling from such an FMM may give a dataset with relatively realistic dependencies, of the type that our model should learn. Thus, we learned also FMMs of yeast GCN4 and SWI5 TFs from the MacIsaac et al. dataset [29], and used them as relatively realistic synthetic models (denoted GCN4 FMM and SWI5 FMM). Finally, in order to test the performance of our approach when strong dependencies are present, we created a model with strong dependencies by adding eight double position features to the GCN4 PSSM model (denoted GCN4 PSSM++). We evaluated the learned models by computing the log-likelihood that the learned models assign to a test set of 10,000 unseen TFBSs sampled from the true model, and by computing the Kullback–Leibler (KL) distance between distributions of the true and learned models. The larger the test set likelihood and the smaller the KL distance, the better the reconstruction. We evaluated two specific aspects of our approach: the dependency of the learning on the penalty term free parameter, *α*, and the minimum number of samples needed for learning FMMs. We repeated each experiment setting three times.

We first tested the effect of the penalty term free parameter, *α*, on the quality of the model reconstruction. Intuitively, the larger the value of *α*, the harder it is to learn large weights. Thus, the value of *α* correlates with our prior belief that the model is simple (close to uniform). Therefore, there is a trade off between setting *α* to large values that decrease the risk of overfitting but might miss important features, and setting it to small values that might allow overfitting but also increase the ability to learn features. Since larger input datasets have relatively larger values for the gradients, a given value of α allows more weak features to be learned in large sets as compared to smaller datasets. This effect fits well with the notion of lower noise in larger datasets (see Lee et al. [55] and references within for an exact analysis). To this end, we varied α in the range of 10^−6^ to 100, while using a varied number of 10–1,000 input sequences. The results in the range 10^−1^≤*α*≤10 and 50–1,000 input sequences are shown in Figure S2 in terms of test set likelihood. The results show that in the range tested the best overall reconstruction performance is achieved for *α*≈1. While smaller values tend to allow overfitting, higher values pose harsh constraints on the learned model and learn too few features. Though the effect is stronger for small datasets, *α*≈1 seems to give good performances also for relatively large datasets.

Second, we estimated the minimum number of samples needed for learning FMMs, by sampling different training set sizes in the range of 10–500. In these experiments, we fixed the penalty term free parameter to *α* = 1. As can be seen in Figure 3, in six out of eight cases, our model reconstructs the true model with high accuracy even with a modest number of ∼100 input TFBSs, and reconstructs the true model nearly perfectly with 200 or more samples. For the more complex GCN4 FMM and SWI5 FMM models, although high accuracy is achieved with ∼200 samples, adding ∼200 more samples further improves the model accuracy. As can be seen from the GCN4 and SWI5 PSSM models, although less than 100 samples might cause overfitting, due to under sampling of the distribution space, when the sample number is sufficient no dependencies are learned. As expected, when the true model includes dependencies between positions, our model significantly outperforms the PSSM, in some cases even when only 20 input sites were used. Examining the learned features, we found that for a sample size of 100 or more, only features that appeared in the true model were learned with significant weights. Our results thus show that when we use synthetic data we can successfully learn FMMs, even with a modest setting of 100 input sequences. These numbers are surprisingly small considering the number of parameters examined. Using smaller sets which represent under sampling of the TFBS distribution space or sets that contain noise may lead to overfitting. Therefore, we expect that similar analyses, using a cross validation scheme, on datasets taken from TRANSFAC [56], JASPAR [57] and MacIsaac et al. [29] to be less successful, as most of these datasets are small or contain a considerable amount of noise. Nevertheless, these numbers are far below those for data generated by current genome wide experiments (such as ChIP-chip and ChIP-seq), so our approach is valid for learning TFBS data in realistic settings.

### FMMs Learned from Aligned Transcription Factor Binding Sites Describe Binding Specificities Better Than PSSMs

Having validated our approach on synthetic data, we next applied it to TFBSs data of human TFs. Our goal was to identify whether FMMs can describe the sequence specificities of human TFs better than PSSMs. To that end, we compared FMMs and PSSMs that were learned from the same sets of aligned TF binding sites. We chose three published sets of aligned binding sites sequences of two important human TFs. The first set contains aligned NRSF binding sites published by Johnson et al. [10]. NRSF binds a DNA element called the neuron-restrictive silencer element (NRSE), canonically described as a 21 bp element. Johnson et al. found 1,655 regions that were enriched for such canonical NRSEs in two independent experiments. The two other sets contain aligned predicted CTCF binding sites published by Kim et al. [13]. They mapped CTCF binding sites through a ChIP-chip experiment, and found a 20 bp motif, defined by a PSSM, that appears in 75% of the binding sites. Using this PSSM motif (and by constraining positions 6, 11, 14, and 16 to match the consensus) the authors predicted 31,905 CTCF binding sites, 12,799 of which are conserved in at least one more vertebrate. We will refer to these two sets of predicted sites as “CTCF predicted” and “CTCF predicted conserved” sites, respectively. Clearly, our choice of input sets is not biased in favor of the FMM. The canonical NRSE motif is characterized by a highly informative PSSM and the two CTCF sets were extracted from the genome using an initial PSSM representation, thus may contain a bias towards independence of different positions.

**Figure 4:**
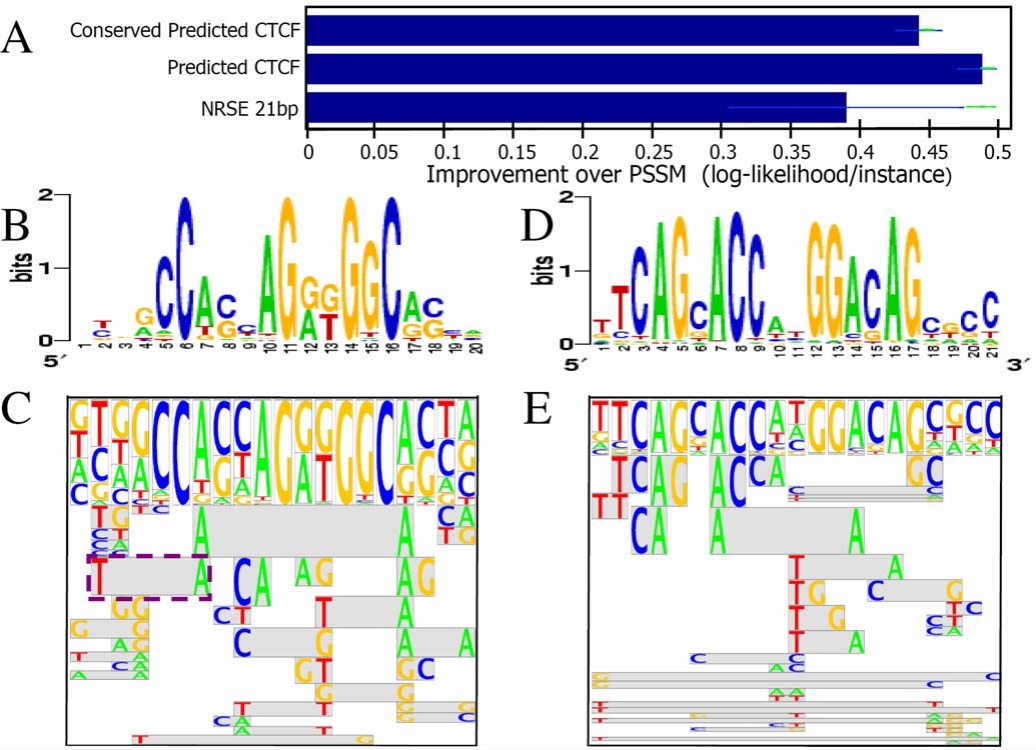
Evaluating our approach on real TFBSs from human. (A) Train (green points) and test log-likelihood (blue bars), shown as the mean and standard deviation improvements in the average log-likelihood per instance compared to a PSSM for the datasets of NRSF, CTCF predicted sites, and CTCF predicted conserved sites. (B) and (C) show the PSSM and FMM features expectations logo for CTCF predicted conserved sites respectively. (D) and (E) show the same for NRSF sites. Each feature in the FMM feature expectation logo ((B) and (E)) is represented by a box. The horizontal position and the letters in the box define the feature. For example, the feature in the purple dashed box in (C) represent the feature “T at position 2 and A at position 7.” The height of the feature is linear with respect to its expectation in the probability distribution defined by the model. Gray background marks a double position feature.

For each input set we tested whether FMM represents the TFBSs better than PSSM using the following 10-fold cross validation (CV) scheme. Each input set was partitioned into ten subsets. Ten CV groups were created, where in each one a different subset was used as test data, while the other nine were used as training set from which both an FMM and a PSSM were learned. For each CV group, we computed the average likelihood of the test TFBSs according to both the PSSM and FMM, as a measure for the learned sequence model success in representing the binding specificities. The difference between the log average FMM likelihood and the log average PSSM likelihood expresses the improvement of the FMM over the PSSM. The mean and standard deviation for these differences were calculated over the ten CV groups. The results for all three input sets are shown in Figure 4A. The FMM model was found to provide 1.3–1.4-fold more likely representations of the binding specificities of the above TFs than the PSSM, with high significance (above 5 standard deviations over the ten CV groups).

Figure 4B and 4C show the PSSM and FMM features expectations logo for CTCF predicted conserved BSs. Although four positions were forced to match the consensus, our FMM recognizes several inter-position dependencies. We will discuss the details of the CTCF motif at the end of the Results section, where we use unaligned CTCF bound regions from the same work [13] to derive de novo FMM using our motif finder. Figure 4D and 4E show the PSSM and FMM features expectations logo for NRSF. Notably, the FMM found that “T” at position 11 has strong dependencies to the consensus sequence at positions 12–14 and 16. On the other hand, “C” at position 6 prefers either “C” or “G” at position 11. These results show that the improvement of the FMM over the PSSM is due to the representation of inter-position dependencies by the FMM.

### Evaluating the Motif Finder Performance on Yeast Transcription Factors Binding Data

As previously described, our motif finder algorithm consists of two steps. The first step results in a collection of K-mer set Motif Models (KMMs). Each KMM is a set of K-mers that defines an enriched motif, and can be used to extract a set of aligned TFBSs from the input positive sequences (see Methods section). These aligned TFBSs are input to the second step, where a motif model is learned from them, be it a FMM or a PSSM. A question arises, then: do KMMs found by the motif finder represent true motifs? Here we show that the KMMs found by our motif finder indeed represent true motifs, and are comparable in quality to motifs found by common motif finding software.

In order to evaluate our motif finder's performance we chose the dataset of Harbison et al. [17]. Although this dataset contains less information for each TF than more recent experiments that used ChIP followed by tilling array or parallel sequencing technologies, it is the most comprehensive study done for TF–DNA interactions and was used by many motif finding software for performance comparison. This data includes 238 sets of sequences of regions which a TF binds under a specific condition (238 TF-condition sets). The 238 datasets represent 146 TFs in various conditions. For 111 TFs (198 datasets), a motif was published by MacIsaac et al. [29]. The MacIsaac et al. motifs were found by two independent motif finding software which use conservation information and were augmented by the authors using literature-known motifs. These motifs were considered by us as biologically true motifs in order to asses the quality of our motifs. For each of the sets we took the sequences from all of the other 237 sets as a negative set. The total number of sequences in all sets is 6,725, with sets ranging between 10 and 195 sequences. Out of the 238 sets, we discarded those with less than 35 sequences, leaving 123 TF-condition sets, for 78 distinct TFs. The choice of the 35 sequences threshold is discussed in Protocol S1.

**Figure 5:**
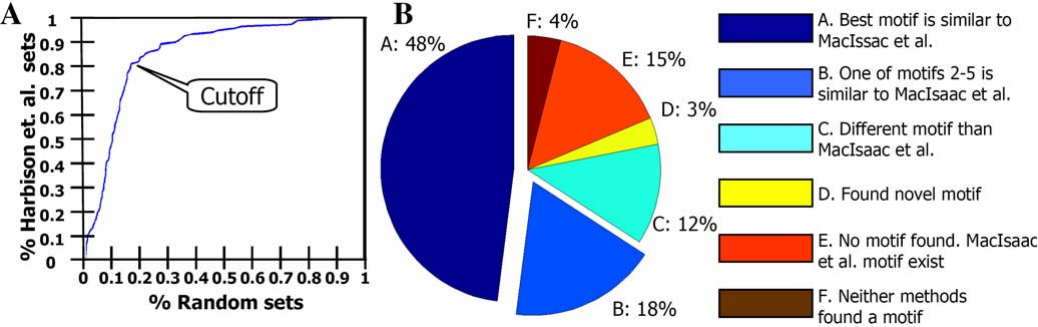
Evaluating the motif finder on yeast data. (A) Shown is the fraction of Harbison et al. [17] sets for which a motif was found with better MHG *p*-value than expected to be found in a set of the same size of randomly selected sequences from Harbison et al. (*x*-axis). We chose a cutoff that defines as a biologically true motif, a motif with MHG *p*-value that is worse than at most 16% of the best motif MHG *p*-value of 1,000 randomly chosen sets. 81% of the Harbison et al. sets contain such a motif. (B) A comparison between KMM motifs predicted by our motif finder and a published combination of predictions by two softwares that use conservation and literature motifs reported in [29].

In order to distinguish between biologically relevant motifs and motifs that can appear by chance, we followed the following procedure. We partitioned the Harbison et al. TF-condition sets into 15 bins according to their sizes. The bins were tagged by the center set sizes, [10,20,…,100,120,…,200]. For example, bin “50” contained all TF-condition sets of sizes 45–54. For each bin “X,” we generated 1,000 sets of X sequences that were randomly picked out of the entire collection of 6,725 Harbison et al. microarray sequences. For each set, all remaining sequences out of the 6,725 sequences were considered as a negative set. We ran our motif finder on all 123 true and 15,000 random sets and computed the best motif MHG *p*-value. We then assessed for each TF-condition set the percentage of random sets in its bin that got a motif with lower MHG *p*-value. We considered this percentage as the empirical *p*-value for getting such MHG *p*-value for a random set. Hence, we considered this empirical test as an assessment of the percentage of false positive motifs for a given MHG *p*-value and a given set size. Figure 5A shows the fraction of TF-condition sets that contain a motif with a MHG *p*-value that is better than the empirical threshold as defined by the *x*-axis for the set bin. It is clear that the results were distinctively better for TF-condition sets than what would be expected by random. As a threshold for biologically relevant motifs we chose for each bin a MHG *p*-value that allowed a random motif finding rate of 16%. At this threshold we found motifs for 81% of the TF-condition sets. Thus, our results suggest that we found true motifs for at least 65% of the TF-condition sets.

Having chosen biologically relevant motif MHG *p*-value thresholds, we next compared the KMM motifs found by our motif finder for the TF-condition sets with the motifs published by MacIsaac et al. [29]. For each TF-condition set, we allowed the motif finder to output up to five unique motifs. Only those that passed their sets' bin threshold were considered. We compared our KMM motifs to the MacIsaac et al. PSSMs by learning a PSSM representation of each KMM, and comparing this PSSM with the respective MacIsaac et al. PSSM, relying on a method previously used by Narlikar et al. [58]. (For a complete description of the motif comparison method, see Protocol S1.) A summary of this comparison is shown in Figure 5B. For 66% of the sets we found motifs similar to those found by MacIsaac et al. These results, although they can only be approximately compared with recently published results by Narlikar et al. [58] and Eden et al. [36], show that our motif finder does not fall behind state of the art motif finders, and is at least comparable to other methods. In Protocol S2, we further compare our motif finder to other motif finders, demonstrating that our motif finder has advantages over other motif finders. Thus, we can use the first step of our motif finder to produce TFBSs data for an FMM-PSSM comparison.

### Learning TF Binding Specificities Features from Unaligned Human and Mouse TF Bound Regions

**Figure 6:**
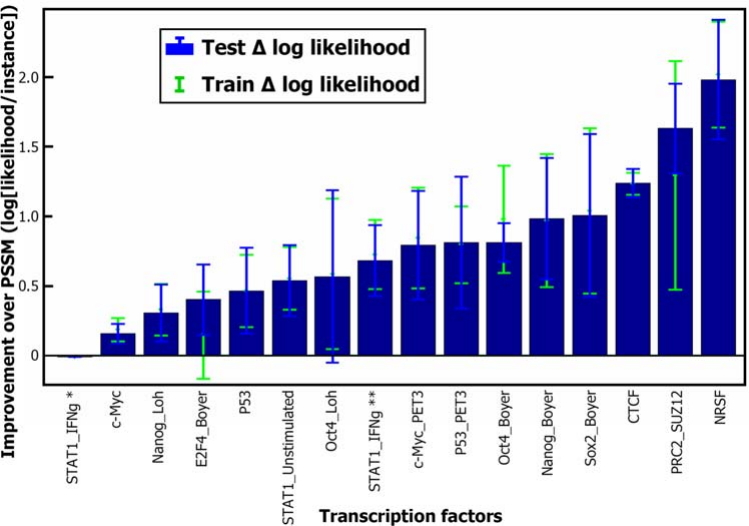
Evaluating our approach on real TFBS enriched sequences datasets from human and mouse. We searched each dataset for de novo motifs using a 5-fold cross validation scheme. We assumed that each sequence in the positive set has at least one TFBS. Following this, we computed for each positive sequence the top motif's FMM and PSSM best TFBS probability and considered it as the sequence binding likelihood. We show here the improvement of our FMM approach over PSSM in terms of train (green dots) and test (blue bars) log average likelihood. In the dataset STAT1_IFNg, two different motifs appear as best/second best in different cross validation runs and are marked by one and two asterisks, respectively.

In a previous section, we compared FMMs and PSSMs learned from aligned TFBS data. Although there are databases that contain sets of aligned TFBS [56],[57], these databases usually contain a relatively small number of TFBS for each TF and contain repeating sequences. Learning FMMs from such datasets, although possible in many cases, might lead to overfitting and is problematic for testing in a cross validation scheme. Recent experiments, however, produced larger sets of TF bound regions. After validating our motif finder on smaller sets, we can now use it to produce aligned TFBS data for a comparison of our FMM approach to the PSSM approach. Table 1 summarizes the collection of datasets that we used. For each dataset, since it contains only positive sequences, we generated a negative set (as described in Protocol S1). We scanned each dataset for de novo motifs in a 5-fold cross validation (CV) scheme. We considered the top motif as the true TF motif. From manual examination of the motifs and comparison of the top motif to the literature, this assumption seems to hold (except for some differences in the Nanog_Boyer and E2F4_Boyer sets motifs). In order to compare the learned FMM to the learned PSSM, we assumed that each sequence in the positive set has at least one TFBS. We computed for each test positive sequence the top motif's FMM and PSSM binding probabilities over all possible locations on the sequence. Following our assumption, for the FMM, as for the PSSM, the best binding probability was considered as the sequence likelihood to be bound by the TF. Figure 6 shows the improvement of our FMM approach over PSSM in terms of test and train log of the average likelihood. The results clearly show that the likelihood of the maximal-likelihood site is better under the FMM model than under the PSSM, and the results are significant in terms of standard deviation over the CV groups. For more than 50% of the sets, the FMM is at least 2-fold more likely to represent the TFs binding specificities. The entire collection of motifs found for all datasets appears in Protocol S2.

**Table 1:** TFBS Enriched Sequences Datasets.

Dataset^a^	Symbol^b^	Enriched protein	Organism	Experiment^c^	Size	Reference
Robertson et al.	STAT1_Unstimulated	STAT1	Human	ChIP-seq	11004	[11]
Robertson et al.	STAT1_INFg	STAT1	Human	ChIP-seq	41582	[11]
Johnson et al.	NRSF	NRSF	Human	ChIPSeq	1946	[10]
Kim et al.	CTCF	CTCF	Human	ChIP-chip	13804	[13]
Lee et al.	PRC2_SUZ12	SUZ12	Human	ChIP-chip	3465	[16]
Wei et al.	P53	P53	Human	ChIP-PET	510	[14]
Wei et al.	P53_PET3	P53	Human	ChIP-PET	307	[14]
Zeller et al.	c-Myc	c-Myc	Human	ChIP-PET	4297	[72]
Zeller et al.	c-Myc_PET3	c-Myc	Human	ChIP-PET	593	[72]
Loh et al.	Oct4_Loh	Oct4	Mouse	ChIP-PET	1051	[15]
Loh et al.	Nanog_Loh	Nanog	Mouse	ChIP-PET	2971	[15]
Boyer et al.	Oct4_Boyer	Oct4	Human	ChIP-microarray	603	[12]
Boyer et al.	Nanog_Boyer	Nanog	Human	ChIP-microarray	1554	[12]
Boyer et al.	Sox2_Boyer	Sox	Human	ChIP-microarray	1165	[12]
Boyer et al.	E2F4_Boyer	E2F4	Human	ChIP-microarray	957	[12]

a Note that the Robertson et al. STAT1 sequences contain two sets: an interferon γ stimulated dataset and unstimulated dataset.

b For p53 and c-MYC we consider both the noisier set of sequences that were represented by two PETs and a smaller and less noisy set (suffixed by “_PET3”) of sequences that were represented by at least three PETs. For every dataset we created a negative dataset as described in Protocol S1.

c Both Chip-seq and ChipSeq (as referred by the authors) use Illumina 1G system as platform. ChIP-PET methodology is described in [14]. ChIP-chip refers to 38 Affymetrix genomewide chips and ChIP-microarray refers to an Agilent promotors microarray.

We focus next on our results for three important human TFs. For the first two, c-Myc and CTCF, we discuss their best FMM and PSSM motifs, and show how their FMM motifs reveal intriguing insights about their binding specificities, that are missed by the PSSM, and that may be correlated with previously published experimental results. For the third, STAT1, we found several motifs, exhibiting the cooccurrence of STAT1 and other TFs binding sites.

#### c-Myc/Max binding specificity features

**Figure 7:**
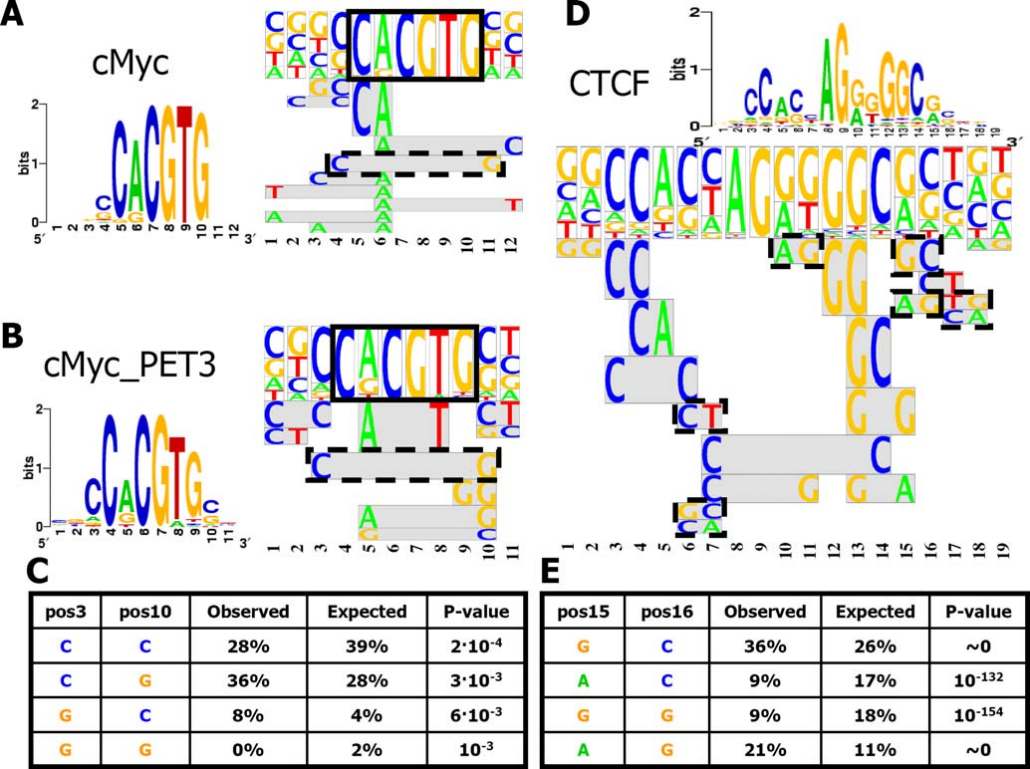
FMM biological findings. (A) c-Myc FMM and PSSM. (B) c-Myc FMM and PSSM learned only from sequences of PET3+ clusters (a cleaner set). The black square in (A) and (B) highlights the E-Box motif. (C) Statistics for the c-Myc FMM feature marked by a dashed line. Expected occurrences are according to the PSSM in (B). The *p*-values were computed using a Binomial test, as described in the Methods section (“Reducing the features space”). (D) CTCF FMM and PSSM. Dashed line squares highlight features that are referenced in the text. (E) Statistics for the CTCF FMM features at positions 15,16 (marked by a dashed line). Expected occurrences are according to the PSSM in (D).

In Figure 7A and 7B we present the FMM and PSSM motifs found by our motif finder, based on unaligned sequences of the datasets “c-Myc” and “c-Myc_PET3” (see Table 1), respectively. Notably, the most significant part of the motif is an E-box motif, marked by a rectangle in Figure 7A and 7B. According to the PSSM in Figure 7A there is only a single low informative position to the left of the E-box. The PSSM in Figure 7B, which relies on less noisy data, adds another low informative position to the right of the E-box. However, when relying on the FMM motifs, even for the noisy data, we find a significant “C-G” feature that connects the two flanking positions of the E-box (and is marked by a dashed rectangle in Figure 7A and 7B). Notably, this feature is palindromic, continuing the E-box palindrome. Comparing the observed dinucleotides at positions 3 and 10 to those expected by single position nucleotide distributions (Figure 7C), reveals a significant enrichment for the “C-G”, and the less abundant “G-C” features. We asked ourselves whether the enrichment of the flanking “C-G” feature by our motif finder is biologically meaningful. We examined the abundance of “C-G” pairs flanking c-Myc/Max canonical E-box (“CACGTG”) hits in the input positive and negative sequence sets of the “c-Myc_PET3” dataset. This pair's relative abundance in the positive set is almost 3-fold higher than in the negative set. The only pair with a higher ratio than that is “C-T” (“C” in position 3 and “T” in position 10), but it appears in a very small number of sequences, less than half the number of sequences in which the “C-G” appeared. We conclude that the FMM captured a potentially important feature that the PSSM misses.

What may be the biological significance of the flanking “C-G” feature? The “CACGTG” E-box is known to be optimal for the binding of not only the c-Myc/Max heterodimer, but also of other basic/helix-loop-helix/leucine zipper (bHLHZ) dimers such as Mad/Max, Max/Max and USF/USF. Past works claimed that flanking bases contribute to binding specificities [59]. In particular, a flanking “C-G” pair was noted to increase binding specificity of c-Myc/Max over Max/Max [60]. Our results support this claim. Moreover, they show how the FMM may reveal biologically important features that reflect different binding modes of a TF.

#### CTCF binding specificities features

In Figure 7D we present the FMM and PSSM motifs found by our motif finder, based on the unaligned sequences of “CTCF” dataset (see Table 1). In a previous section we used TFBSs that were predicted by Kim et al. [13] to learn FMM and PSSM (Figure 4B and 4C). We note that the motifs learned from the predicted TFBS data and those learned from the original unaligned sequences data are highly similar (although several positions in the predicted sites were constrained to match the consensus). In particular, when comparing the two FMMs, we find similar dinucleotide features. We note especially the dinucleotide features connecting the neighboring positions 6–7, 10–11, 15–16, and 17–18 in Figure 7D (marked by dashed rectangles) that correspond to features in positions 8–9, 12–13, 17–18, and 19–20 in Figure 4C. In both FMMs, we find a significant 12 bp core (positions 3–14 in Figure 7D and 5–16 in Figure 4C). Indeed, this 12 bp core element was recently experimentally identified by Renda et al. [61] as essential for high affinity binding of CTCF. Notably, the FMM finds mostly dinucleotide features that are contained within each of the four triplets that comprise the core, with little sequence diversity in the two outer triplets, relative to the inner triplets (as exhibited by the features in positions 6–7 and 10–11 in Figure 7D and 8–9 and 12–13 in Figure 4C that do not follow the consensus in these positions). This also corresponds to the findings of Renda et al. that the core is bound by 4 out of the 11 zinc finger (ZF) domains of the CTCF, where the two ZFs that bind the outer triplets play a more important role in the binding. The confinement of dinucleotide features within triplets seem to break outside of the 12 bp core, as exhibited by the features in positions 15–16 and 17–18 in Figure 7D and 17–18 and 19–20 in Figure 4C. Not much is known about the CTCF binding to the regions flanking the 12 bp core. Our FMMs suggest sequence features that are important for the weaker and less specific binding to these regions.

Notably, our results are also well correlated with recently published work by Xie et al. [62], who found three variants of CTCF binding motifs (represented by PSSMs) that are highly conserved in mammalian genomes. The main differences between these three variants correspond to the dinucleotide features found in positions 6–7 and 15–16 in Figure 7D and 8–9 and 17–18 in Figure 4C, emphasizing that the FMM detects features that are involved in partitioning the TFBS space into subclasses.

Finally, to emphasize the significance of features captured by the FMM in Figure 7D, the table in Figure 7E compares observed numbers of dinucleotides at positions 15 and 16 in TFBSs found by our motif finder, to those expected by single position nucleotide distributions. Thus, FMMs capture several candidate features that define important CTCF binding specificities.

#### STAT1 motifs

We ran our motif finder on two STAT1 datasets. The “STAT1_IFNg” (see Table 1) set includes sequences bound by STAT1 in human HeLa S3 cells stimulated by IFNγ, while the “STAT1_Unstimulated” set includes sequences bound by STAT1 in unstimulated cells. In the IFNγ stimulated data, we found a highly enriched GAS motif (the third best motif), that was not found in the unstimulated data, as expected from the literature (see [11]). Interestingly, we found that the AP1 motif, “TGAGTCA,” is the best motif for the IFNγ stimulated data and the third best motif for the unstimulated data. This result supports the recent results of Bhinge et al. [63], who point at the significance of STAT1-AP1 binding sites' cooccurrence. Further, we found a GC-rich motif as the best motif for the unstimulated data and the second best for the IFNγ stimulated data. This GC-rich motif resembles the GC-box motif to which SP1 binds. Cooccurrence of STAT1 binding sites and GC-boxes and STAT1-SP1 interaction have been previously reported for specific promotors [64]. Our findings suggest a genomewide role for the STAT1-SP1 interaction in both conditions. The AP1 and the GC-rich motifs are the ones for which the cross validation testing results appear in Figure 6 (the AP1 motif is the one marked by an asterisk). These results demonstrate our motif finder's ability to extract multiple meaningful motifs from a single input dataset.

## Discussion

In this paper we present *feature motif models* (FMMs), a novel probabilistic method for modeling the binding specificities of TFs. The current dominant model used is the position specific scoring matrix (PSSM), which assumes independence between binding motif positions. The richer FMM formulation may be viewed as a generalization of the PSSM model, enabling complex position dependencies to be captured, as well as other high-level features (e.g., palindromes). In this work, we used FMM models that extend the PSSM by capturing dinucleotide dependencies.

To show that FMM models describe TF binding motifs better than PSSM models, we compared their likelihoods over held-out synthetic and real data. The real biological data included both aligned TFBS data and unaligned TF bound regions data. We showed that for all types of data, the FMM representation of motifs outperforms the PSSM representation.

Importantly, FMMs can be presented using a clear and easy to understand logo, where important position dependencies are plainly visible. Examining our FMM results for two human TFs, c-Myc and CTCF, we found intriguing dinucleotide features that may be important for their binding. Some of those features are well-correlated with previously published results, while others may provide hypotheses on the binding specificities of these TFs. These hypotheses can be further studied experimentally to gain better understanding of how the TF recognizes its binding sites. Notably, in those examples, the FMMs hint at the importance of positions that are regarded uninformative by the PSSMs.

In order to allow de novo FMM motif finding, we developed a novel motif finder. Our motif finder finds motifs that are discriminatively enriched in a positive set of unaligned sequences over a negative set of unaligned sequences. For each motif, it learns either an FMM or a PSSM representation. An important property of our motif finding algorithm is that it extracts enriched sets of *K*-mers from the data, thus maintaining dependencies between positions, if such exist. We show that our motif finder performs well and use it to demonstrate how FMMs can easily be integrated as a basic building block of a motif finding software which represents TF binding specificities. As a future direction, we suggest to integrate the FMM into both common and novel state-of-the-art motif finding algorithms.

We demonstrated the benefits of using log-linear models (a representation of Markov networks) for representing important features of TF binding specificities, and suggested a methodology to learn such features from both aligned and unaligned input sequences. In the Methods section we also contribute to the general problem of learning log-linear models by suggesting a methodology for optimizing the objective function, which may give better performance under settings that require approximation.

There are several directions for refining and extending our FMM approach. First, our rich framework can model many other types of features. Examples of features that can be added are: to what extent is the sequence a palindrome and the structural curvature of the sequence. Another direction is to add to our learning process the ability to learn binding energies associated with a given set of sequences. Finally, using our models as an improved basic building block, we can integrate it into higher level regulatory models (e.g., [65]) and obtain a much better quantitative understanding of the underlying principles of transcriptional regulatory networks.

## Methods

### The Feature Motif Model

We now present our approach for representing TF binding specificities. Much like in the PSSM representation, our goal is to represent commonalities among the different TFBSs that a given TF can recognize, and assign a different strength to each potential site, corresponding to the affinity that the TF has for it. The key difference between our approach and the PSSM is that we want to represent more expressive types of motif commonalities compared to the PSSM representation, in which motif commonalities can only be represented separately for each position of the motif. Intuitively, we think of a TF–DNA interaction as one that can be described by a set of sequence *features*, such as pairs or triplets of nucleotides at key positions, which are important for the interaction to take place: the more important features a specific site has, the higher affinity it will have for the TF.

One way to achieve the above task is to represent a probability distribution over the set of all sequences of the length recognized by the given TF. That is, for a motif of length *L*, we represent a probability distribution over all 4*^L^* possible *L*-mer sequences. Formally, we wish to represent a joint probability distribution *P*(*X*_1_,…,*X_L_*), where *X_i_* is a random variable with domain {A,C,G,T} corresponding to the nucleotide at the *i*th position of the sequence. However, rather than representing this distribution using the prohibitively large number of 4*^L^*−1 independent parameters, our goal is to represent this joint distribution more compactly in a way that requires many fewer parameters but still captures the essence of TF–DNA interactions. The PSSM does exactly this, but it forces the form of the joint distribution to be decomposable by positions. Barash et al. [46] presented alternative representations to the PSSM, using Bayesian networks, that allow for dependencies to exist across the motif positions. However, as discussed above, the use of Bayesian networks imposes unnecessary restrictions and is not natural in this context.

A more natural approach that can easily capture our above desiderata is the framework of undirected graphical models, such as log-linear representation of Markov networks (log-linear model), which have been used successfully in an increasingly large number of settings. As it is more intuitive for our setting, we focus our presentation on log-linear models. Let *X* = {*X*_1_,…,*X_L_*} be a set of discrete-valued random variables. A *log-linear model* is a compact representation of a probability distribution over assignments to *X*. The model is defined in terms of a set of *feature functions f_k_*(*X_k_*), each of which is a function that defines a numerical value for each assignment *x_k_* to some subset *X_k_* ⊂ *X*. Given a set of feature *functions F* = {*F_k_*}, the parameters of the log-linear model are weights *θ* = {*θ_k_*: *f_k_* ∈ *F*}. The overall joint distribution is then defined as:
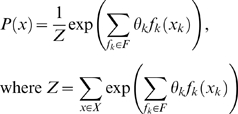
(1)is the *partition function* that ensures that the distribution *P* is properly normalized (i.e., ∑*_x_*_∈*X*_ *P*(*x*) = 1), and *x_k_* is the assignment to *X_k_* in *x*. Although we chose the log-linear model representation, we note that it is in fact equivalent to the Markov network representation, and the mapping between the two is straightforward. We now demonstrate how we can use this log-linear model representation in our setting, to represent feature-based motifs. We start by showing how PSSMs can be represented within this framework.

#### Representing PSSMs

Recall that a PSSM defines independent probability distributions over each of the *L* positions of the motif. To represent PSSMs in our model, we define 4 features *f_iJ_* for each position that indicate whether a specific nucleotide *J* ∈ {A,G,C,T} exists at a specific position 1≤*i*≤*L* of the TFBS. We associate each feature with a weight *θ_iJ_* that is equal to its marginal log probability over all possible TFBSs. It is easy to show that putting this into Equation 1 defines the exact same probability distribution as of the PSSM, and that the *partition function* as defined in Equation 1 is equal to 1 in this case.

#### Representing feature motifs

Given a TF that recognizes TFBSs of length *L*, our feature-based model represents its motif using the log-linear model of Equation 1, where each feature *f_k_* corresponds to a sequence property that may be defined over multiple positions. As an example for a feature, consider the indicator function: “C” at position 2 and “G” at position 3, as in Figure 1. This feature illustrates our ability to define features over multiple positions. Although in this work we focus on indicators of a single nucleotide or a nucleotide pair, we note that continuous and even global features (such as G/C content) can easily be defined within our model. We then associate each feature with a weight, *θ_k_*, that defines its importance to the TF–DNA binding affinity. Given a sequence, we can now compute its probability using Equation 1, which boils down to summing the value of all the features present in the sequence, each multiplied by its respective weight parameter, and exponentiating and normalizing this resulting sum. Intuitively, this model corresponds to identifying which of the features that are important for the TF–DNA interaction are present in the sequence, and summing their contributions to obtain the overall affinity of the TF to the site. This intuitive model is precisely the one we set out to obtain.

### Learning Feature Motif Models

In the previous section, we presented our feature-based model for representing motifs. Given a collection of features *F*, our method uses the log-linear model to integrate them, as in Equation 1. As we showed, the standard PSSM model can be represented in our framework. However, our motivation in defining the model was to allow for integration of other features, which may span multiple positions. A key question is how to select the set of features for a given model. In this section, we address this problem. Since log-linear models are equivalent to Markov networks, our problem essentially reduces to structure learning in Markov networks. This problem is quite difficult, since even the simpler problem of estimating the parameters of a fixed model does not have an analytical closed form solution. Thus, the solutions proposed for this problem have been various heuristic searches, which incrementally modify the model by adding and deleting features to it in some predefined scheme [55],[66].

We now present our algorithm for learning a feature-based model from TFBSs data. Our approach follows the Markov network structure learning method of Lee et al. [55]. It incrementally introduces (or selects) features using the *grafting* method of Perkins et al. [54]. We first present the simpler task of estimating the parameters of a given model, as this is a sub-problem that we need to solve when searching over the space of possible network structures.

#### Parameter estimation

For the parameter estimation task, we assume that we are given as input a dataset *D* = {*x*[1],…,*x*[*N*]} of *N aligned* i.i.d TFBSs, each of length *L*, and a model *M* defined by a set of sequence features *F* = {*f*_1_,…,*f_k_*}. Our goal is to find the parameter vector *θ* = {*θ*_1_,…,*θ_k_*} that specifies a weight for each feature *f_i_* ∈ *F*, and maximizes the log-likelihood function
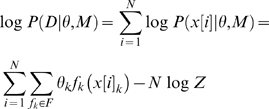
(2)where *x*[*i*]*_k_* corresponds to the nucleotides of the *i*th TFBS at the positions relevant to feature *k*, and *Z* is the partition function as in Equation 1. It can easily be shown that the gradient of Equation 2 is
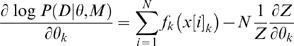
(3)


Although no closed-form solution exists for finding the parameters that maximize Equation 2, the objective function is concave (as discussed by Lee et al. [55]), and we can thus find the optimal parameter settings using numerical optimization procedures such as gradient ascent or conjugate gradient [67]. We now deal with optimizing Equation 2.

#### Optimization of the objective function

Applying numerical optimization procedures such as gradient ascent requires the computation of the objective function and the gradient with respect to any of the *θ_k_* parameters. Although the fact that the objective function is concave and that both the function and its gradient have simple closed forms may make the parameter estimation task look simple, in practice computing them may be quite expensive. The reason is that the second terms of both the function and the gradient involve evaluating the partition function, which requires, in a naive implementation, summing over 4*^L^* possible TFBSs sequences.

Since algorithms for learning log-linear models usually require computation of the partition function, this problem was intensively researched. Although in some cases the structure of the features may be such that we can decompose the computation to achieve efficient computation, in the general case it can be shown to be a NP-hard problem and hence requires approximation. Here we suggest a novel strategy of optimizing the objective function. We first use the (known) observation that the gradient of Equation 2 can also be expressed in terms of features expectations. Specifically, since
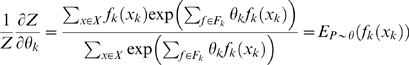
(4)we can rewrite Equation 3 as:
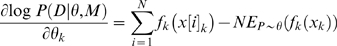
(5)


We further observe that since Equation 2 is a concave function, its absolute directional derivative along any given line in its domain is also a concave function. We used this observation to use the conjugate gradient function optimization algorithm [67] in a slightly modified version: Although the gradient that was given to the algorithm was indeed as in Equation 5, the function value along every line search step of the algorithm was the absolute directional derivative along this line. For example, at the line search step along direction *y* our function F*(*θ*,*y*) value is: F*(*θ*,*y*) = |〈▿ log *P*(*D*|*θ*,*M*)〉|. Following the above strategy allows us to optimize Equation 2 without computing its actual value. Specifically, it means that we can optimize our objective without computing the partition function. Instead, the problem reduces to evaluating feature expectations, a special case of inference in log-linear models, that can be exactly computed using algorithms such as *loopy belief propagation* [68]. The ability of these algorithms to give an exact result depends on the underlying network structure. As the network structure becomes more complex, the algorithms need to use approximations. Since this family of algorithms can also approximate the partition function, our method will be similar to methods that evaluate the partition function when the network structure allows for exact inference. However, as the error bounds for approximate inference are better characterized then the error bounds of partition function estimations, it is possible that our approach may work better under conditions that require approximation.

### Learning the Features

Above, we developed our approach for estimating the feature parameters for a fixed model in which the feature set *F* is defined. We now turn to the more complex problem of automatically learning the set of features from aligned TFBSs data. This problem is an instance of the more general problem of learning the structure of Markov networks from data. However, quite surprisingly, although Markov networks are used in a wide variety of applications, there are very few effective algorithms for learning Markov network structure from data.

In this paper we followed the Markov network structure learning approach suggested by Lee et al. [55]. This approach extends the learning approach of Perkins et al. [54] to learning the structure of log-linear representation of Markov networks using the *L*_1_-Regularization over the model parameters. To incorporate the *L*_1_-Regularization into our model we need to introduce a *Laplacian* parameter prior over each feature, leading to the modified objective function:
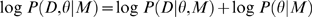
(6)where 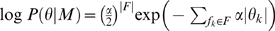 and log *P*(*D*|*θ*,*M*) is the data likelihood function as in Equation 2. Taking the log of this parameter prior and eliminating constant terms, we arrive at the final form of our objective function:
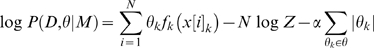
(7)


It is easy to see that this modified objective function is also concave (as it is an addition of a concave function and a linear function) in the feature parameters *θ* and we can thus optimize it using the same conjugate gradient procedure described above. We then follow the *grafting* approach of adding features in a stepwise manner. In each step, the algorithm first optimizes the objective function relative to the current set of active features *F*, and then adds the inactive feature *f_i_* ∉ *F* with the maximal gradient at *θ_i_* = 0. Using an *L*_1_-Regularized concave function provides a stopping criteria to the algorithm that leads to the global optimum [54]. The *L*_1_-Regularization has yet another desirable quality for our purpose, as it has a preference for learning sparse models with a limited number of features [55]. It has long been known to have a tendency towards learning sparse models, in which many of the parameters have weight zero [69] and theoretical results show that it is useful in selecting the features that are most relevant to the learning task [70]. Since the *grafting* feature addition method is a heuristic, it seems reasonable that features that were added at an early stage may become irrelevant at later stages, and hence get a zero weight. We thus introduce an important difference from the method of Lee et al., by allowing the removal of features that become irrelevant.

#### Reducing the features space

Although the method described above is complete in the sense that it searches over all possible features for the features that are relevant for the optimal solution, the number of possible motifs increases with the max size of the feature domain *D* and the length of the motif *L* (*O*(*L^D^*)). For these reasons we incorporated a preprocessing step that reduces the space of possible features by considering only features that pass a statistical test for significance. In this work we used a Binomial test to evaluate the statistical significant of a feature *f* of the form “nucleotide 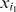 at position *i*_1_ and nucleotide 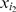 at position *i*_2_” that appear in *n* TFBSs out of *N* TFBSs. The null hypothesis is that the two positions are independent, and therefore the test p-value is calculated as follows: 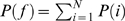 where 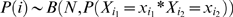. We control for multiple hypothesis false positive using FDR [53] (with a threshold of 0.2). As a future improvement for our algorithm we can incorporate diverse statistical tests in this step such as those suggested by Tomovic et al. [71].

### Finding De Novo FMM Motifs

In the previous sections we described how to learn an FMM model from aligned TFBS data. We now turn to the more complex problem of finding de novo FMM elements that are enriched in a target set of relatively long and unaligned sequences compared with a background set. Recent years have shown a development of several high throughput methods reviewed in the introduction. The most dominant methods include chromatin immunoprecipitation (ChIP) of DNA-bound proteins followed by either DNA chip (ChIP-chip) [12],[13],[16] or high throughput sequencing (ChIP-PET, ChIPSeq/ChIP-seq) [10],[11]. The common analysis of these two methods usually includes a step of peak finding (or fitting) [10],[21] which results in a set of unaligned DNA sequences that are bounded by the TF with a measure of intensity. A common practice is then to define an intensity cutoff. This cutoff defines a target set of sequences bound by the TF (positive set), while the sequences below some cutoffs are defined as background set (negative set). The problem of optimally determining such a cutoff was previously addressed by Eden et al. [48]. We, however, do not address it, and assume that the cutoff is given. Having these two sets we can search for a TF binding motif that is enriched in the positive set compared to some negative model, or compared to the negative sequences set. Here we developed a novel motif finder that searches the positive set for motif elements that are enriched compared to the negative set. Our motif finder is unique in its ability to output either PSSM or an FMM. We use the motif finder both to produce data for an FMM - PSSM comparison, and as a proof of concept of integrating FMM into a motif finding algorithm.

To properly describe our motif finding algorithm, we introduce the notion of a *K*-mer set motif model (KMM). A KMM consists of a set of short aligned (not necessarily overlapping) sequences (see Figure S1 for a KMM example). The KMM can be described as having an “OR” term between all of its sequences. Following this description, a “hit” of a KMM is defined as an appearance (“hit”) of at least one of its sequences in an input sequence. Our motif finding algorithm consists of two main steps. In the first step we extract KMMs that are enriched in the positive set compared to the negative set. In the second step we use the hits of each of the top scored KMM in the positive set to generate aligned sequences (these are putative TFBSs) from which a FMM or a PSMM is learned.

In the first step we start with extracting the hits of all sequences of length *K* (referred as “*K*-mers”) that appear in positive or negative input sequences (Figure 2 (1)). In order to evaluate the enrichment of a *K*-mer (and later a KMM) in the positive set compared to the negative set, we use its hits count as input for the multidimensional hypergeometric *p*-value (MHG *p*-value) test, introduced by Eden et al. [36]. Preferring the MHG *p*-value over a “simple” hypergeometric *p*-value has the benefit of quantifying the significance of a multiple motif occurrence in the same sequence in a data driven manner [36]. As Eden et al., we too restricted ourselves to three dimensions, considering cases of 0, 1, and ≥2 hits per input sequence. Let *n* be the number of positive sequences, and *N* be the total number of sequences (positive and negative). For a certain KMM, suppose first that there is a single KMM hit in *K*_1_ of the *N* sequences and in *k*_1_ of the *n* positive sequences, and second, that there are two or more KMM hits in *K*_2_ of the *N* and in *k*_2_ of the *n* positive. The multidimensional hypergeometric probability for that event is given by:
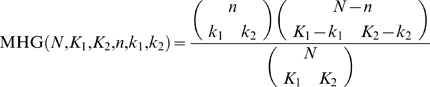
and the multidimensional hypergeometric *p*-value is given by:
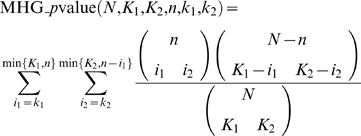



Next we rank the *K*-mers based on their MHG *p*-value (Figure 2 (2)). We use a *p*-value threshold MHG_threshold_, controlled by FDR (in this work we used MHG_threshold_ = 10^−3^), to filter the *K*-mers (Figure 2 (3)). Due to computational resources constraints, we limit the maximum number of *K*-mers that pass the filter to *T* = 200 top scored *K*-mers. We use the filtered *K*-mers to construct a “*K*-mer graph” (Figure 2 (4)). Each *K*-mer forms a singleton KMM and is a vertex of the graph. Two *K*-mers (vertices) are connected by an undirected edge if and only if one of two conditions hold: either the Hamming distance between the *K*-mers does not exceed a threshold *H*_Distance_ (in this work we used *H*_Distance_ = 1), or the two *K*-mers can be perfectly aligned when one is shifted up to *M*_Shift_ base pairs with respect to the other (in this work we used*M*_Shift_ = 1).

The node with the best MHG *p*-value is then chosen as a “seed KMM”, and a greedy algorithm performs a series of steps along the edges in which the seed neighbors are joined into the seed KMM vertex, growing its KMM (Figure 2 (5)). For a detailed description, see Protocol S1. In brief, this step either adds the neighbor *K*-mer to the seed KMM or uses the neighbor *K*-mer to extend a subset of the seed KMM sequences (this step allows the KMM to grow in length). Each time, the step that best improves the KMM seed's MHG *p*-value is chosen. When no such step exists, the seed node is removed from the graph and a new seed node is chosen, repeating the process of growing the seed KMM. When there are no more nodes left in the graph, the KMMs are ranked by their MHG *p*-value, and the best *M* are picked (Figure 2 (5)). The above process is repeated for every *K*_min_≤*K*≤*K*_max_ (in this work we used *K*_min_ = 5 and *K*_max_ = 8). At the end of the first step the best *M* of all picked KMMs are chosen as input for the next step.

In the second step, the motif finder produces either a PSSM or a FMM for each KMM. For each KMM, the algorithm uses all of its hits in the positive set to generate aligned TFBS data (Figure 2 (6)), with the length of these TFBSs (which will be the motif length) induced by the KMM alignment (for an elaborate illustration of this process see Figure S1). It then learns the requested model (FMM or PSSM) that describes the KMM hits. As a last step similar KMM motifs are removed (those with larger MHG *p*-value) and unique motifs are outputted (the similarity measure is described in Protocol S1).

A special case that our motif finder recognizes and handles is that of dimer motifs. KMMs may represent dimers by holding two different alignment offsets per single K-mer sequence. For a detailed description of how dimer motifs are recognized and produced, see Protocol S1.

The main novelty in our motif finder is in its ability to produce FMMs instead of PSSMs. Producing FMMs requires the motif finding algorithm to preserve inter-position dependencies, if they exists in the data. Our KMM methodology of producing motifs from *K*-mers, and of properly extracting TFBSs that contain these *K*-mers from the data, ensures that we learn a motif model from TFBSs in which inter-position dependencies are indeed preserved.

Finally, the performance of our motif finder with respect to memory and running time is discussed in Protocol S1.

## Supporting Information

Figure S1 An example for a transition from KMM to FMM or PSSM. The KMM in this example contains four short sequences. The length of the KMM sequence alignment is 11 bp. Hence, we determine that the motif length will be 11 bp long. We next extract all of the hits of each of the KMM K-mers in the positive set. We extend each hit of a K-mer according to the KMM alignment to produce an 11 bp long putative TFBS. For example, for “Seq1” hits we extend two bases to the left and one to the right, due to its position in the alignment. Note that different K-mers may have mutual hits (in the figure the sequence is surrounded by a blue dashed line is a hit for both “Seq2” or “Seq3”). In this way we generate a set of 11 bp long aligned putative TFBS sequences from which we can learn an FMM or PSSM.
(0.77 MB TIF)

Figure S2 Evaluation of the L1 penalty term free parameter on synthetic data. FMM model performance in terms of the average test set likelihood on eight synthetic datasets (sampled from the models in Figure 3) as a function of the number of data instances and the L1 penalty free parameter ((alpha)). We observed that the effect of the value of (alpha) is, as predicted, much stronger on small datasets. Where too small values of (alpha) might not prevent overfitting (those resulting in low average test likelihood), too large values might pose too harsh restriction on the learned features. However, relatively small values of (alpha) ((alpha) = 1) have prevented overfitting for PSSM sampled datasets of size 1,000. On the basis of these results, we selected the value 1, which gave relatively good performances on all datasets, for our runs.
(11.12 MB TIF)

Protocol S1 Supporting methods.
(0.77 MB PDF)

Protocol S2 Supporting results.
(11.12 MB PDF)
