# Supplementary figures and images for: A Feature-Based Approach to Modeling Protein–DNA Interactions
Source: PLoS Comput Biol. 2008 Aug 22;4(8):e1000154. doi: 10.1371/journal.pcbi.1000154 (PMC2516605; doi:10.1371/journal.pcbi.1000154)

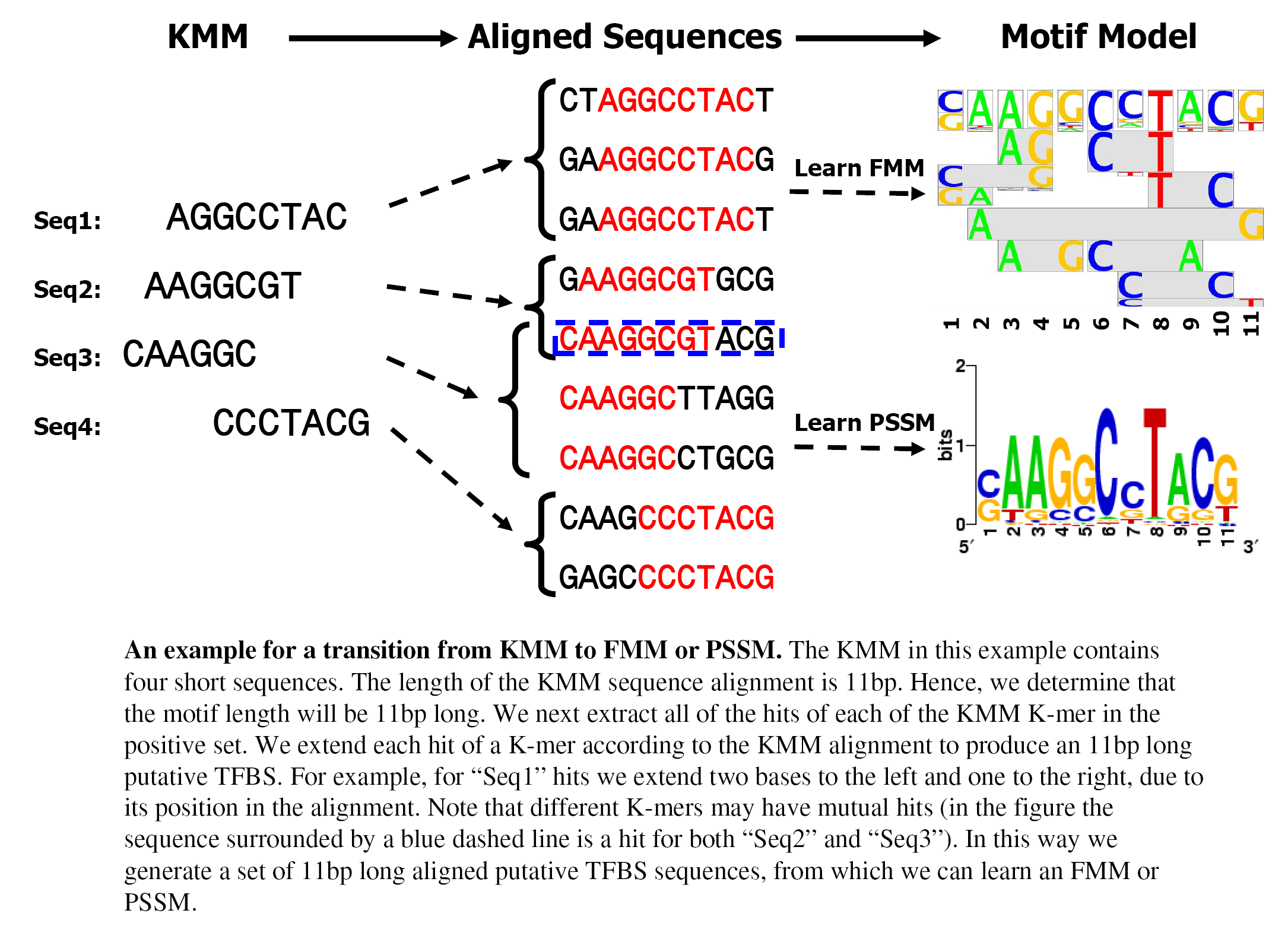

Supplement: Figure S1 — An example for a transition from KMM to FMM or PSSM. The KMM in this example contains four short sequences. The length of the KMM sequence alignment is 11 bp. Hence, we determine that the motif length will be 11 bp long. We next extract all of the hits of each of the KMM K-mers in the positive set. We extend each hit of a K-mer according to the KMM alignment to produce an 11 bp long putative TFBS. For example, for “Seq1” hits we extend two bases to the left and one to the right, due to its position in the alignment. Note that different K-mers may have mutual hits (in the figure the sequence is surrounded by a blue dashed line is a hit for both “Seq2” or “Seq3”). In this way we generate a set of 11 bp long aligned putative TFBS sequences from which we can learn an FMM or PSSM. (0.77 MB TIF) [file pcbi.1000154.s001.tif]

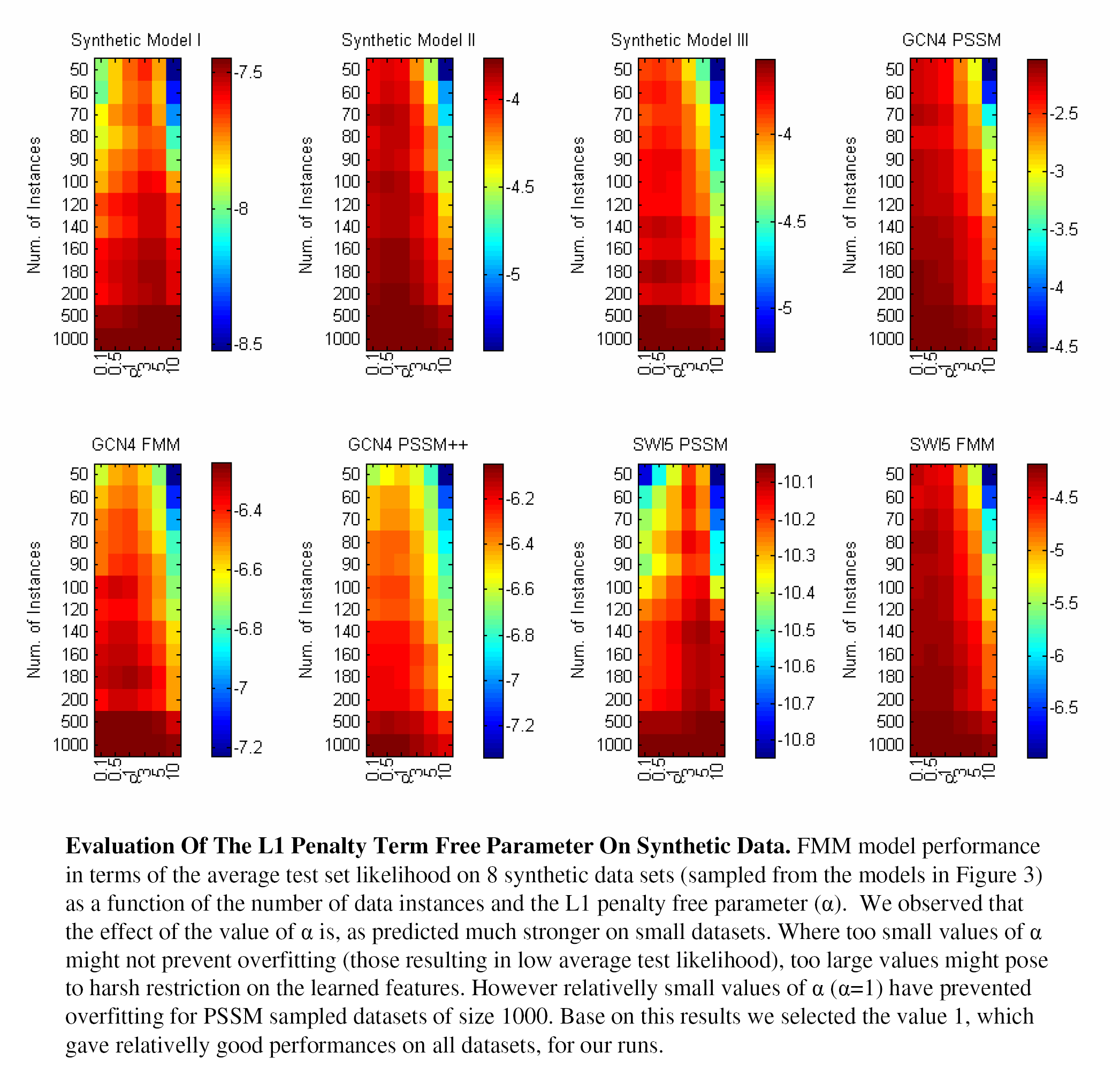

Supplement: Figure S2 — Evaluation of the L1 penalty term free parameter on synthetic data. FMM model performance in terms of the average test set likelihood on eight synthetic datasets (sampled from the models in Figure 3) as a function of the number of data instances and the L1 penalty free parameter ((alpha)). We observed that the effect of the value of (alpha) is, as predicted, much stronger on small datasets. Where too small values of (alpha) might not prevent overfitting (those resulting in low average test likelihood), too large values might pose too harsh restriction on the learned features. However, relatively small values of (alpha) ((alpha) = 1) have prevented overfitting for PSSM sampled datasets of size 1,000. On the basis of these results, we selected the value 1, which gave relatively good performances on all datasets, for our runs. (11.12 MB TIF) [file pcbi.1000154.s002.tif]
