# Supplementary material for: A Feature-Based Approach to Modeling Protein–DNA Interactions
Source: PLoS Comput Biol. 2008 Aug 22;4(8):e1000154. doi: 10.1371/journal.pcbi.1000154 (PMC2516605; doi:10.1371/journal.pcbi.1000154)
Supplement: Protocol S1 — Supporting methods. (0.77 MB PDF) [file pcbi.1000154.s003.pdf]

Supplemental Methods

A KMM Example. Here we elucidate the definition of the KMM and the process by which it is used to generate a motif model - either a PSSM or a FMM. **Figure S1** illustrates an example of a KMM (on the left side) that contains four sequences. As illustrated, the sequences are properly aligned in a manner that preserves their position relative to the KMM motif (as described in the following section). In order to transform the KMM into a motif model we extract all of the hits of each of the KMM K-mer sequences in the positive sequences input set. We extend each hit of a K-mer according to the KMM alignment. We then use this set of extended hits as a set of aligned TFBSs from which we learn either a PSSM or a FMM. This transition from KMM to a motif model is used both during the KMM growing algorithm to maintain a proper alignment (as described below in **Maintaining the KMM Alignment**), and in the final step of creating a motif model from the KMM hits in the positive sequences set.

Growing the Seed KMM. After its construction the K-mer graph contains a singleton K-mer in each vertex. The vertex with the best MHG *p*-value is chosen as a "seed KMM". Next, a greedy algorithm is used to "grow" the KMM set and improve its MHG *p*-value. The algorithm performs a series of steps along the edges of the graph in which neighbors of the seed KMM are joined into it. Before we describe the KMM growing algorithm, let us refine the definition of a KMM. A KMM contains two sets of sequences: an active set $S = \{s_1, ..., s_{N_S}\}$ and a "non-contributing" set $T = \{t_1, ..., t_{N_T}\}$. While the active set defines the output KMM and contribute to the MHG *p*-value computation, the "non-contributing" does not, and is used only during the KMM growing process. A hit (appearance in an unaligned input sequence) of the KMM is thus defined as a hit of at least one of $s_i \in S$. At initiation the seed KMM is defined by $S = \{s_1\}$ and $T = \{\}$ (empty set).

We will now describe a step of the greedy KMM growing algorithm. Let us assume, without loss of generality, that at a certain point during the algorithm's

run, the seed KMM contains *N* active sequences $S=\{s_1,\ldots,s_N\}$ and *M* non-contributing sequences $T=\{t_1,\ldots,t_M\}$. Assume we consider joining a neighbor vertex to the seed KMM. As mentioned above, this vertex will contain a singleton KMM. We will refer to the single sequence in the neighbor KMM as *s*'.

The algorithm can perform two types of join steps: an "OR" step and an "And" step. In an "OR" step, *s*' will simply be added to the seed node KMM's active set, such that the KMM will then contain the active set $S=\{s_1,\ldots,s_N,s'\}$. In an "AND" step, the algorithm merges *s*' with a subset of the KMM sequences $V=\{s_{i_1},\ldots,s_{i_k},t_{j_1},\ldots,t_{j_l}\}$. A merge operation means that each $v_i \in V$ can be perfectly aligned to *s*' when one is shifted up to M_{Shift} base pairs with respect to the other. The merge operation creates a new sequence that is the concatenation of the first sequence with the unaligned remainder of the other sequence. Denote the new sequence set generated by merging *s*' with each of the sequences of *V* as *V*'. An "AND" step as defined above will result in the KMM with the active set $(S\setminus\{s_{i_1},\ldots,s_{i_k}\}\cup V')$ and the non-contributing set $(T\setminus\{t_{j_1},\ldots,t_{j_l}\}\cup V)$. This explains the role of the non-contributing sequences for future "AND" steps.

Before taking the next step, the algorithm considers all possible "OR" and "AND" steps. Each step is evaluated in terms of the seed KMM's MHG *p*-value after it. The step that achieves the best *p*-value is chosen and the chosen neighbor is joined into the seed KMM. When no step that improves the seed KMM's MHG *p*-value exists, the seed KMM vertex is removed from the graph and a new seed KMM vertex is chosen. The algorithm repeats the above process iteratively until there are no more vertices left in the graph. The algorithm then ranks all the outputted KMMs by their MHG *p*-value, and the best *M* are chosen as output for the next phase of the algorithm.

Maintaining the KMM Alignment. Suppose we perform a step (an "OR" step or an "AND" step) on the graph, resulting in the addition of a new sequence *s*' to the KMM active set *S*. In order to maintain a proper alignment of the new active set, we find the offset of *s*' with respect to *S*'s alignment prior to the step. In order to

do so, we first use the hits of *S* in the positive input set to learn a motif (in this work we learn a PSSM), denoted by *P* (see **Figure S1** for illustration). This motif represents the KMM before the step. We then use *P* to scan around each hit of *s*' in the positive set, and for each offset between *P* and the hit we compute the log probability of the subsequence that is aligned with *P*. We then sum for every offset the log probabilities computed over all the hits of *s*'. The offset with the maximum sum of log probabilities is chosen as *s*' KMM alignment offset.

Determining Similarity of Motifs - KMMs Similarity. In order to remove similar KMMs from the final output of the first step of the motif finding algorithm (see **Figure 2**) we need to identify similar motifs. We will now describe the method we use to determine similarity of KMMs. Given two KMMs, we compare each sequence of the first KMM to each of the sequences of the second KMM. Suppose sequences s_1 and s_2 are compared. Denote by L_1 and L_2 the sequences lengths, respectively, and let $L_{\min} = \min(L_1, L_2, L_{\min\,align}^{KMM})$ (in this work we used $L_{\min\,align}^{KMM} = 6$). Sequences s_1 and s_2 are regarded as similar if s_2 or s_2's reverse complement can be perfectly aligned with a shifted s_1, such that the aligned part's length is at least $L_{\min\,align}^{KMM}$. The two KMMs are regarded as similar if a sequence of the first and a sequence of the second are found to be similar. We used this method to remove similar de-novo found motifs (see **Figure 2**).

Determining Similarity of Motifs - KMM Similarity to PSSM. In order to evaluate the performance of our motif finder we wanted to compare its output to known TF motifs. Since known TF motifs are usually represented as PSSMs, we decided to use the PSSM output version of our algorithm. Given an output KMM we first used the algorithm standard procedure to learn a PSSM, which we will refer to as P^K (see **Figure S1**). Assume, we want to compare our motif (P^K) to a known motif (P). Denote by L_K and L_P the two PSSMs lengths respectively, and let $L_{\min} = \min(L_P, L_K, L_{\min\,align}^{PSSM})$ (in this work we used $L_{\min\,align}^{PSSM} = 6$). To compare the

two PSSMs, we used the Inter-Motif distance suggested by by Harbison *et al.* [1] in a similar way to Narlikar *et al.* [2]. This measure defines the distance between two PSSMs under a given alignment as follows:

$$D(P,P^{K}) = \frac{1}{L\sqrt{2}} \sum_{i=1}^{L} \sum_{b\in\{A,C,G,T\}} (P_{i,b} - P_{i,b}^{K})^{2}$$

where L is the aligned part length. Let us now denote by $A = \{a_1,\ldots,a_j\}$ the set of all partial alignments of P and P^K of length $L \le L_{\min}$, such that the aligned part of P^K has an average information content of at least one bit. We also denote by $D_i(P,P^K)$ the distance between P and P^K under a specific alignment a_i. We can now define the measure of similarity between the two PSSMs, $dist(P,P^K)$, by the equation:

$$dist(P,P^{K}) = \max_{a_i\in A}\{D_i(P,P^{K})\}$$

As in Narlikar *et al.*, P and P^K are considered similar if $dist(P,P^K) \le 0.25$. In that case, the KMM and P are considered to be similar.

Recognizing Dimer Motifs. A special case that our motif finder recognizes and handles, is that of dimer motifs. Testing whether a motif is a dimer motif is performed right after a new seed KMM vertex is chosen, containing a single K-mer. First, a PSSM that represents the seed KMM K-mer is learned (**Figure S3** (a)). We add uniform prior pseudo counts to refrain from zero probabilities. Next, the PSSM is used to “scan” the surroundings of each hit of the seed K-mer in the positive sequences set, computing the log probability of the sequence at each offset relative to the K-mer hit according to the PSSM (Suppl. **Figure S3** (B)). The PSSM log probabilities are summed for every offset over all the seed K-mer hits. Clearly, for every K-mer hit the highest probability will be at offset 0, (exactly at the seed K-mer hit). However, in the case of a dimer motif, that has a signal repeat of length *R* (where *R* = monomer length + length of gap between

monomers), we assume that the seed K-mer reside within a monomer. Hence, a similar K-mer is expected to appear in the other monomer, at an offset of *+R* or *-R* relative to the hit. So for the dimer case, the second and third best offsets overall will be the *±R* offsets (as illustrated at **Figure S3** (B)). This test might fail in cases where the K-mer length is larger than *R*, or where the K-mer length is much smaller than *R* and we do not scan far enough around the seed K-mer hits. These cases, however, can be overcome by choosing a reasonable range of K-mer lengths, and a large enough range of scanning. False positives may be of less concern, due to the fact that our algorithm will generate both dimer and monomer motifs, and so false "dimer" motifs will most likely be discarded later.

Growing Dimer KMMs. KMMs may represent dimers by holding two different alignment offsets per single KMM sequence. When growing a dimer KMM, each new sequence is passed through the dimer recognition test (same as described above for the first K-mer). If it passes the test, then it receives two alignment offsets, one for each monomer. Else, it simply receives the best alignment offset found for it. Note that each KMM sequence donates all of its hits to the KMM, even if they do not necessarily fall within dimer binding sites, hence the KMM enrichment is the monomer enrichment in the data.

Learning Dimer Motifs. Since the dimer KMM consists essentially of monomer sequences, when we wish to generate aligned dimer sequences (and from them learn a FMM or a PSSM) as described in **Figure S1** we may introduce a lot of noise, in the form of non dimeric sequences that contain only a single monomer. Such sequences may be added for two reasons: First, the input data sequences contain not only dimer binding sites but also monomer binding sites (whether those are biologically valid sites, we do not claim). Second, even for hits in true dimer binding sites, since for a KMM sequence we do not know in advance for any of its hits whether it occurs in the left or right monomer, for each hit we take both surrounding putative dimer sequences. **Figure S4** describes the procedure we perform in order to generate an alignment of dimer binding site sequences: An

initial PSSM representation of the dimer KMM motif, of length L_D, is learned (**Figure S4** (A)), as described in **Figure S1**. This PSSM model is based on aligned sequences that suffer from both noise origins mentioned above. This initial dimer PSSM is used (**Figure S4** (B)) to scan the surroundings of every hit of each KMM sequence (represented by a short blue line in **Figure S4** (B)) in the positive sequences set, computing the log probability of each sequence of length L_D along the scan. The one that gets the maximal log probability (represented by a golden line in **Figure S4** (B)) is picked. This process rids of noise of the second origin, as described above. However (**Figure S4** (C)), the noise from the first origin (monomer binding sites) remains, along with true dimer binding sites (surrounded by a light green rectangle in **Figure S4**). In order to be able to asses whether a sequence contains a dimer or only a monomer, a monomer KMM is produced (**Figure S4** (D)) from the dimer KMM (by properly taking only a single offset per KMM sequence), and a PSSM representing the monomer KMM, of length L_M, is learned. This monomer PSSM is used (**Figure S4** (E)) for scanning each of the above picked L_D long sequences. For dimer binding site sequences the offset between the two best hits is expected to be exactly *R*, the signal repeat. Sequences for which the offset between two best hits was not *R* are discarded (**Figure S4** (F)). The remaining are aligned and used as TFBSs from which a FMM or PSSM representing the dimer motif will be learned.

Motif Finder Running-Time and Accepted Input Data Size. Our motif finder accepts large sequence data sets as input, with memory consumption scaling approximately linearly with their size. However, the running time does not necessarily scale linearly with the input size. For different data sets, K-mer grpahs with different levels of sparseness may be generated during the run. As the graph is less sparse, running time may scale quadratically. The level of sparseness depends mainly on underlying properties of motifs enriched in the data, and not on the set size. The following examples relate to run time on a machine with 2.6GHz processor and 4GB RAM. As a first example, for a data set of 152 positive sequences and 513 negative sequences, of mean length 338

bases, the run time was 10min, with 4 K-mer graph run iterations (with seed lengths ranging between 5 and 8 bases). As a second example, for a data set of 1872 positive sequences and 3744 negative sequences of mean length 810 bases, the run time was 3hr:23min, with 4 K-mer graph run iterations (with seed lengths ranging between 5 and 8 bases). As a third example, for a data set of 15000 positive sequences and 15000 negative sequences of mean length 1069, the run time was 10hr:42min, with a single K-mer graph run iteration (with a seed length of 8 bases). Currently, on a 4GB RAM machine, our motif finder copes with ~35000 input sequences of mean length 1000.

Choice of a Set Size Threshold for the Motif Finder Performance Evaluation. In the motif finder evaluation section we described how we generated motifs for random sets from the Harbison *et al.* data, in order to compare their MHG *p*-values with those of motifs found for the Harbison *et al.* TF-condition sets. Each TF-condition set was compared with the random sets of closest size among 15 different sizes ([10,20…100,120…200]). In **Figure S5** we present the distributions of log MHG *p*-values for the TF-condition sets, partitioned into different bins by the compared random sets of the same size (for example, the 47 TF-condition sets of sizes 15 to 24 sequences were compared to the random sets of size 20). As can be seen, for very small sets, of less than 35 positive sequences, motifs found by our motif finder could not be effectively distinguished from motifs that appear in randomly selected sets of the same size. On the contrary, for sets larger than 35, distinction was good. In general, it is evident that as set size increases, motifs found by our motif finder in true TF-condition sets are rarely random motifs. Hence, we performed the motif finder evaluation on sets of size 35 or more.

Generating Negative Sets. Each data set contained positive sequences only. We generated negative sets by taking for each positive sequence the two flanking sequences of same length. For the "E2F4_Boyer" and the "PRC2_SUZ12" sets, since they had high GC content, we generated a negative set by randomly extracting for each positive sequence a sequence of same length and similar GC content (±5%) from the human genome. For each set we used only sequences shorter than 3000 bases. For the "STAT1_IFNg" set we randomly reduced the positive and negative set sizes to 15,000 sequences each (because of run time memory constraints, see the the motif finder run-time performance analysis section).

Generating FMM Sufficient Statistics. The first step of learning an FMM from aligned TFBS dataset, is to collect sufficient statistics for the set of all possible features. In this work we focus on features that are indicators for the appearance of specific nucleotides in a specific set of positions (for example: "A at position 2, and G at position 4"). Therefore, the set of all possible features is defined as the collection of such features over all possible combination of motif positions and nucleotides. The size of the set of all possible features, F, can be bound using the inequality: $|F| \le \sum_{d=1}^{D} \prod_{i=0}^{d-1} A(L-i) = O(A^D L^D)$ where A is the alphabet size (for DNA $A = 4$), D is the maximum domain size and L is the motif length. Although this number can get quite large (for example: $A = 4, D = 2, L = 20 \Rightarrow |F| \le 6160$ and $A = 4, D = 3, L = 20 \Rightarrow |F| \le 443920$), usually a TFBS data set contains only a small fraction of all possible features. The sufficient statistic for each feature is defined as the number of appearances of the feature in the data set. This number, divided by the size of the aligned TFBS dataset, is equal to the empirical probability of the feature. This probability is used in later steps of the algorithm including statistical tests that can reduce the feature pool size. The first step of learning an FMM from an aligned dataset of binding sites, is to count the appearances of all the features that exist in the data set.

Figures

Figure S1

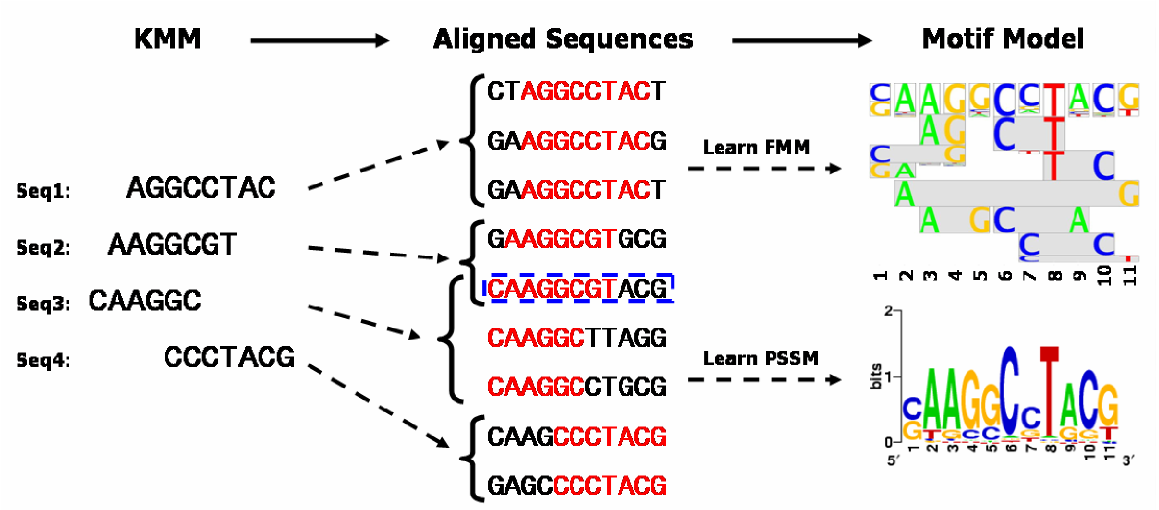

An example for a transition from KMM to FMM or PSSM. The KMM in this example contains four short sequences. The length of the KMM sequence alignment is 11bp. Hence, we determine that the motif length will be 11bp long. We next extract all of the hits of each of the KMM K-mer in the positive set. We extend each hit of a K-mer according to the KMM alignment to produce an 11bp long putative TFBS. For example, for “Seq1” hits we extend two bases to the left and one to the right, due to its position in the alignment. Note that different K-mers may have mutual hits (in the figure the sequence surrounded by a blue dashed line is a hit for both “Seq2” and “Seq3”). In this way we generate a set of 11bp long aligned putative TFBS sequences, from which we can learn an FMM or PSSM.

Figure S3

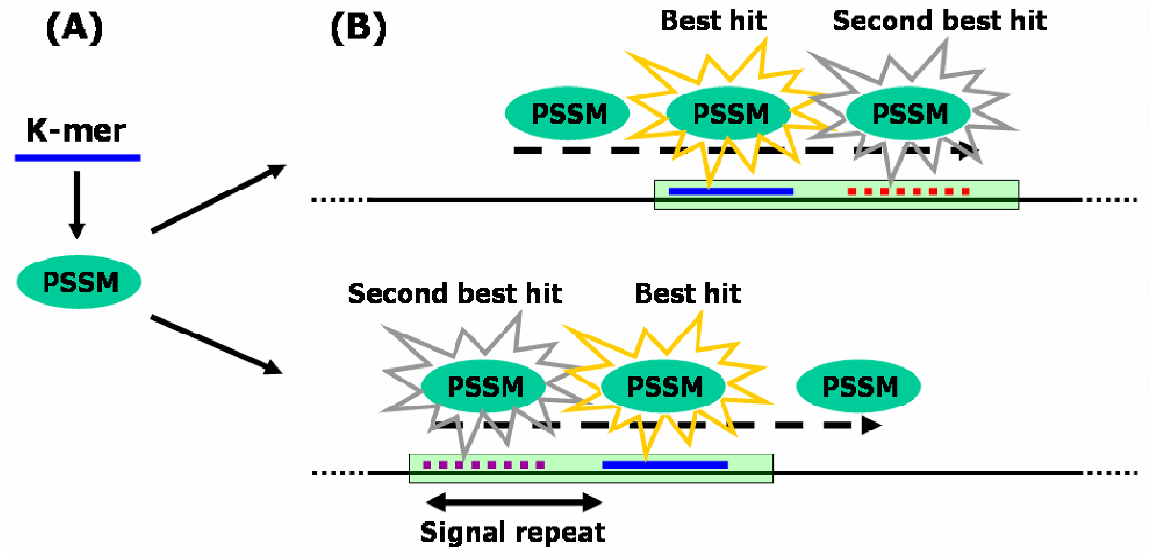

Testing for a dimer. (A) A PSSM that represents the seed KMM K-mer is learned and used to scan (B) the K-mer hits surrounding area. Here a K-mer that is part of a monomer of a dimer motif is illustrated. Hence each K-mer hit has another K-mer hit in a fixed $\pm R$ offset.

Figure S4

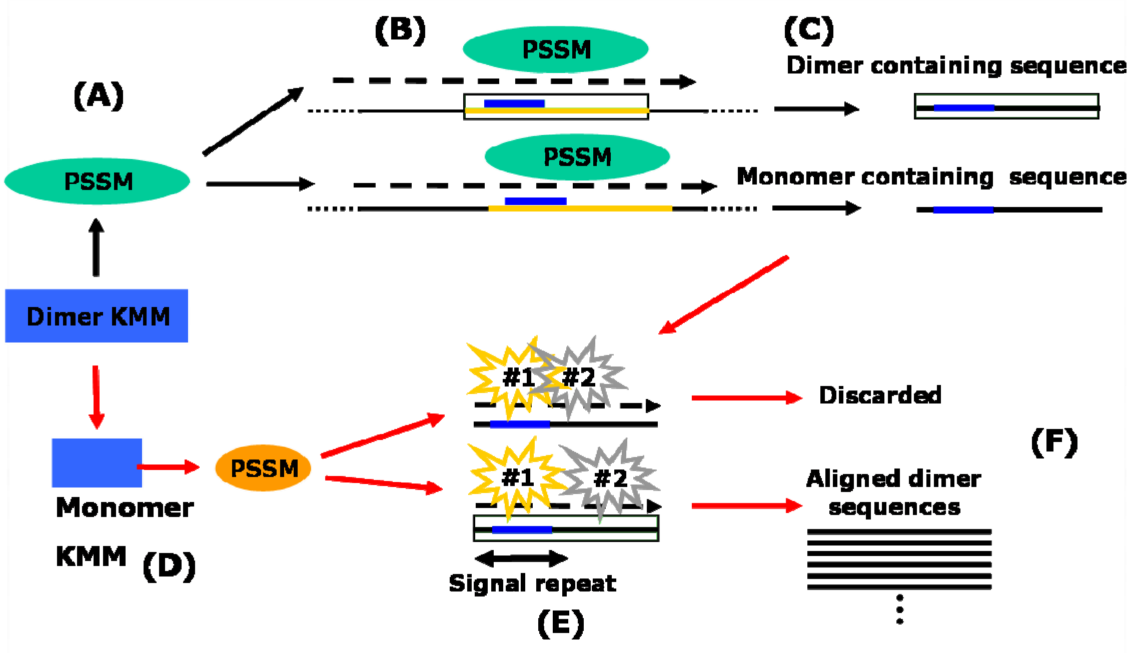

Generating an alignment of dimer binding site sequences. (A) An initial PSSM representation of the dimer KMM motif is learned from an alignment that contains both true dimer sequences and false ones. (B) The dimer PSSM is used to build a set of dimer sequence candidates with a higher ratio of true dimer sequences. (C) Still, this set contains both sequences that are from dimer binding sites (in light green rectangle) and such that are not (contain monomer binding sites) and need to be discarded. (D) A monomer KMM and its PSSM representation are generated. (E) The monomer PSSM is used to scan each of the sequences picked in (B). For dimer binding site sequences (in light green rectangle) the offset between the two best hits is expected to be exactly *R*, the signal repeat. (F) Sequences for which the offset between two best hits was not *R* are discarded. The remaining are regarded as dimer binding site sequences.

Figure S5

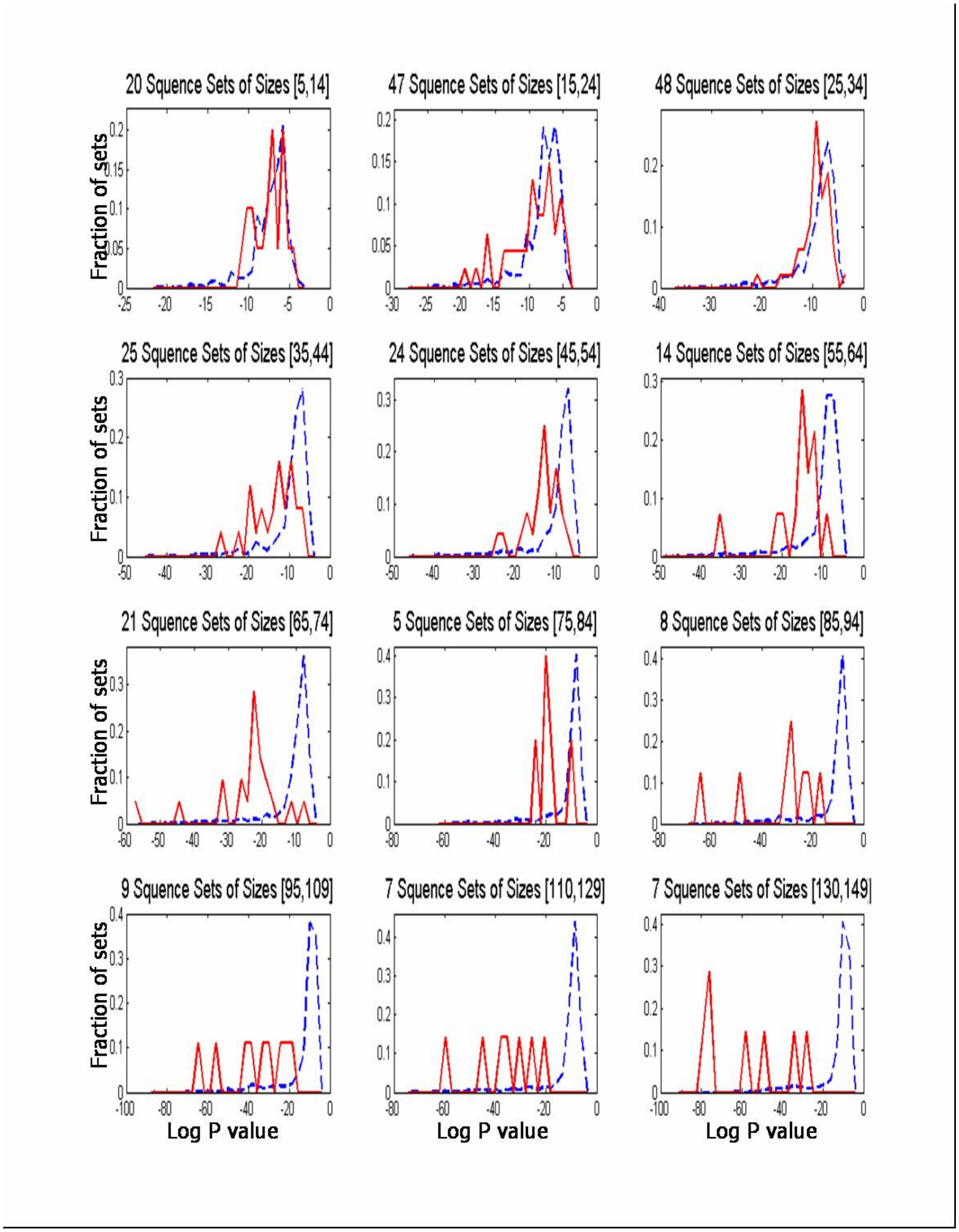

Comparison of Harbison *et al.* TF-condition sets and random sets MHG *p*-values. MHG *p*-values distributions for binned groups of Harbison *et al.* TF-condition sets (in red) and for the respectively sized randomly selected sets (in blue). The title of each plot defines range of sets size and the number of TF-condition sets in the bin.

References

1. Harbison CT, Gordon DB, Lee TI, Rinaldi NJ, Macisaac KD, et al. (2004) Transcriptional regulatory code of a eukaryotic genome. Nature 431: 99-104.
2. Narlikar L, Gordan R, Hartemink AJ (2007) A nucleosome-guided map of transcription factor binding sites in yeast. PLoS Comput Biol 3: e215.
