# Supplementary material for: A Feature-Based Approach to Modeling Protein–DNA Interactions
Source: PLoS Comput Biol. 2008 Aug 22;4(8):e1000154. doi: 10.1371/journal.pcbi.1000154 (PMC2516605; doi:10.1371/journal.pcbi.1000154)
Supplement: Protocol S2 — Supporting results. (11.12 MB PDF) [file pcbi.1000154.s004.pdf]

Supplemental Results

Comparing our FMM Motif Finder to Other Available Motif Finders. We sought to test our motif finder's performance versus other available motif finder software. A variety of such tools exists, and the question arises: What are the benefits of using our motif finder, as FMM motif models can be learned from aligned TFBSs that are extracted from the data by any other motif finder. As data, we chose most of the datasets described in Table 1: *NRSF, CTCF, P53_PET3, c-Myc_PET3, Oct4_Loh, Nanog_Loh, Oct4_Boyer, Nanog_Boyer, Sox2_Boyer*. For each set, we used the 5-fold cross validation (CV) scheme that we used in section "*Results: Learning TF Binding Specificities Features from Unaligned Human and Mouse TF Bound Regions*".

For each dataset, we used the following protocol:

(1) For each CV group, and for each motif finder: run with the training data as input, and acquire putative aligned TFBSs for the best motif.

(2) For each CV group, and for each motif finder: learn both a FMM and a PSSM representation of the best motif, from the aligned TFBSs generated in step 1.

(3) For each CV group, and for each motif finder: score each of the test sequences (positive and negative) by the log-likelihood of the best hit of the FMM in that sequence (*FMM_score*), and similarly with the PSSM (*PSSM_score*, so each sequence has two scores). (Repeat also for the train sequences).

(4) For each CV group, and for each motif finder: rank all test sequences (positive and negative) by their *FMM_score* (from highest to lowest). Using ROC (receiver operator characteristic) analysis, based on the above ranking, calculate the AUC (the area under the ROC curve), as a measure of how well the FMM discriminates the positive set from the negative set (an AUC of 0.5 is no better than random, the higher the AUC the better the discrimination). Call this AUC *FMM_AUC* and use it as a score of the FMM. Similarly, calculate the *PSSM_AUC*. (Repeat also for the train sequences).

(5) For each motif finder other than our FMM motif finder: for each CV group, calculate the differences: "FMM_AUC(FMM motif finder) - FMM_AUC(other motif

finder)", "PSSM_AUC(FMM motif finder) - PSSM_AUC(other motif finder)", "FMM_AUC(FMM motif finder) - PSSM_AUC(other motif finder)". Calculate the means and standard deviations of the above differences over the five CV groups. (Here the protocol ends).

To follow the above protocol, we sought to compare our motif finder to other motif finders that output aligned TFBSs (as required by step 1). Different motif finders may find motifs of different lengths, thus they cannot be compared directly based on the likelihood of their best hits (*FMM_score* and *PSSM_score*). Since we expect a true motif to discriminate between the positive and negative sets, we chose the AUC score as a basis for comparison. This score eliminates the fear of motif-length related bias in favor of any of the motif finders. As we used a discriminative score, and as our FMM motif finder is discriminative, we sought to test our motif finder also versus a discriminative motif finder. To meet the above, we compared our motif finder's performance with three other: *AlignACE* [1], *MDscan* [2] and *DEME* [3]. The first two are non discriminative, thus were run using only the positive training sequences sets as input. The last is a state-of-the-art discriminative motif finder, thus received the negative training sequences sets as well (as did our own motif finder). The three motif finders were run with default parameters. *MDscan* and *DEME* require that the motif width be given as input. For that matter we used the lengths of the best motifs found by our motif finder for the datasets (see below in "*Supplemental Results: De-Novo Motifs*"). The comparison results are summarized in **Figures S6-S8**.

Figure S6 shows the results when we compared PSSMs learned by our motif finder to PSSMs learned by the other tools. In a majority of the cases, our PSSMs were found to better represent the motif. This supports the claim that our motif finder does not produce aligned TFBSs that are wrongfully biased against the PSSM representation.

Figure S7 shows the results when we compared FMMs learned by our motif finder to PSSMs learned by the other tools. In a majority of the cases our FMMs were found to better represent the motif. This supports our basic claim that producing FMM motif models using our motif finder has an advantage over the

PSSMs that other motif finders produce.

Figure S8 shows the results when we compared FMMs learned by our motif finder to FMMs learned by the other tools. In a majority of cases our FMMs were found to better represent the motif. This demonstrates the advantage of using our motif finder in order to learn FMM motif models.

Figure S6

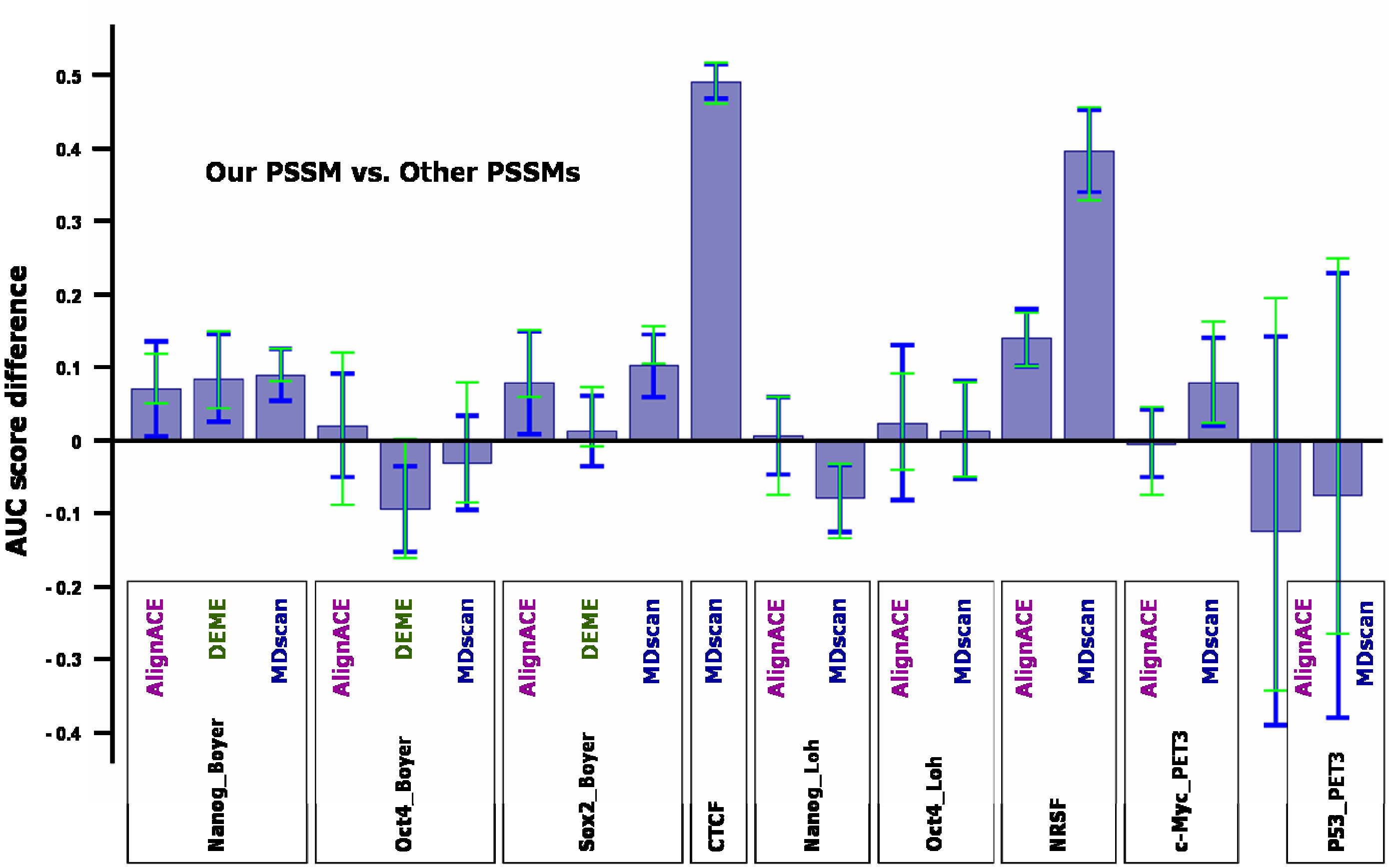

Differences between AUC Scores of our PSSMs and the other motif finders PSSMs. The means and the standard deviations (calculated over the five CV groups) of the difference between our PSSM AUC score and the other tool PSSM AUC score. The blue bars and error bars are for AUC scores based on the test data. The light green error bars are for AUC scores based on the train data.

Figure S7

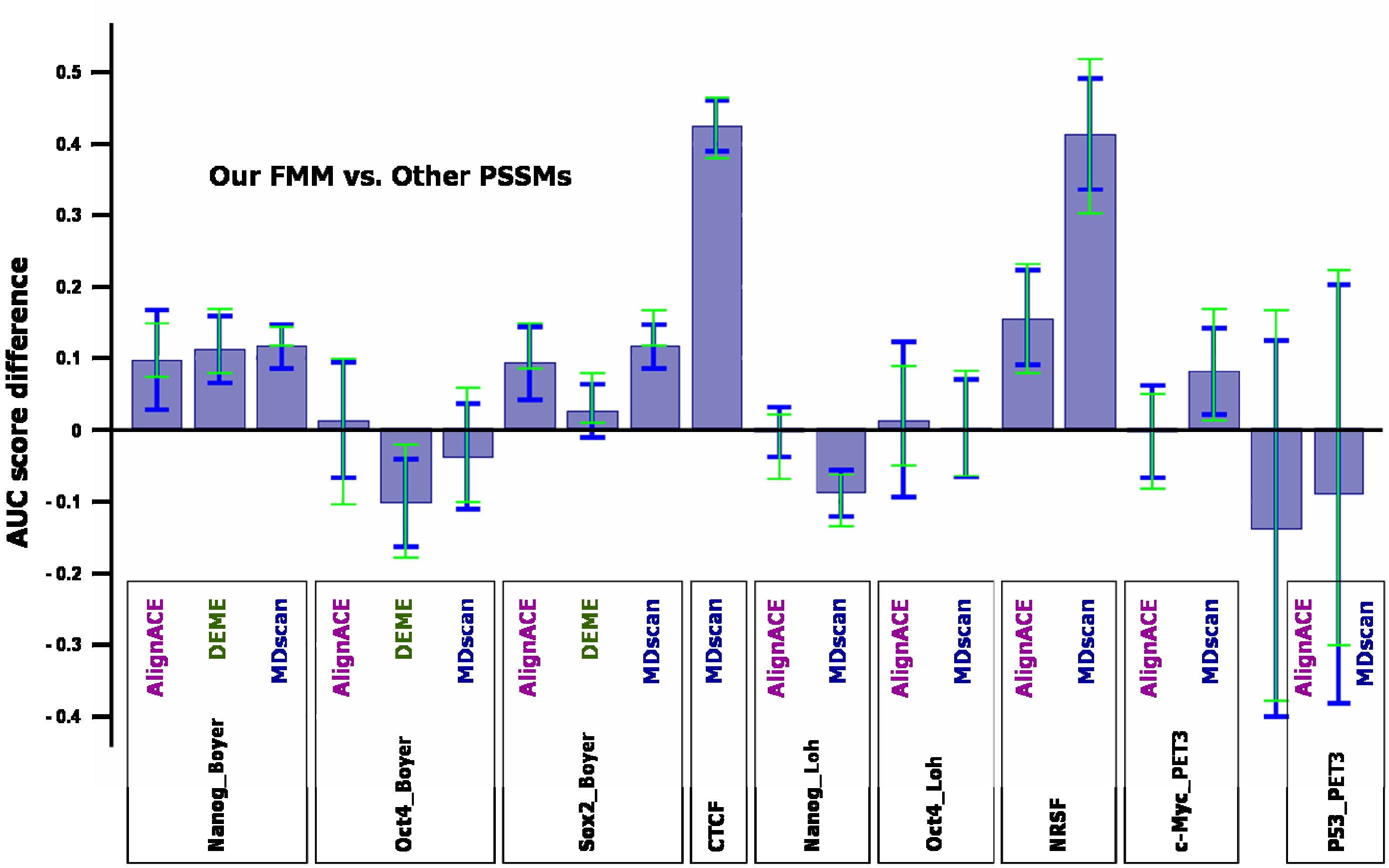

Differences between AUC Scores of our FMMs and the other motif finders PSSMs. The means and the standard deviations (calculated over the five CV groups) of the difference between our FMM AUC score and the other tool PSSM AUC score. The blue bars and error bars are for AUC scores based on the test data. The light green error bars are for AUC scores based on the train data.

Figure S8

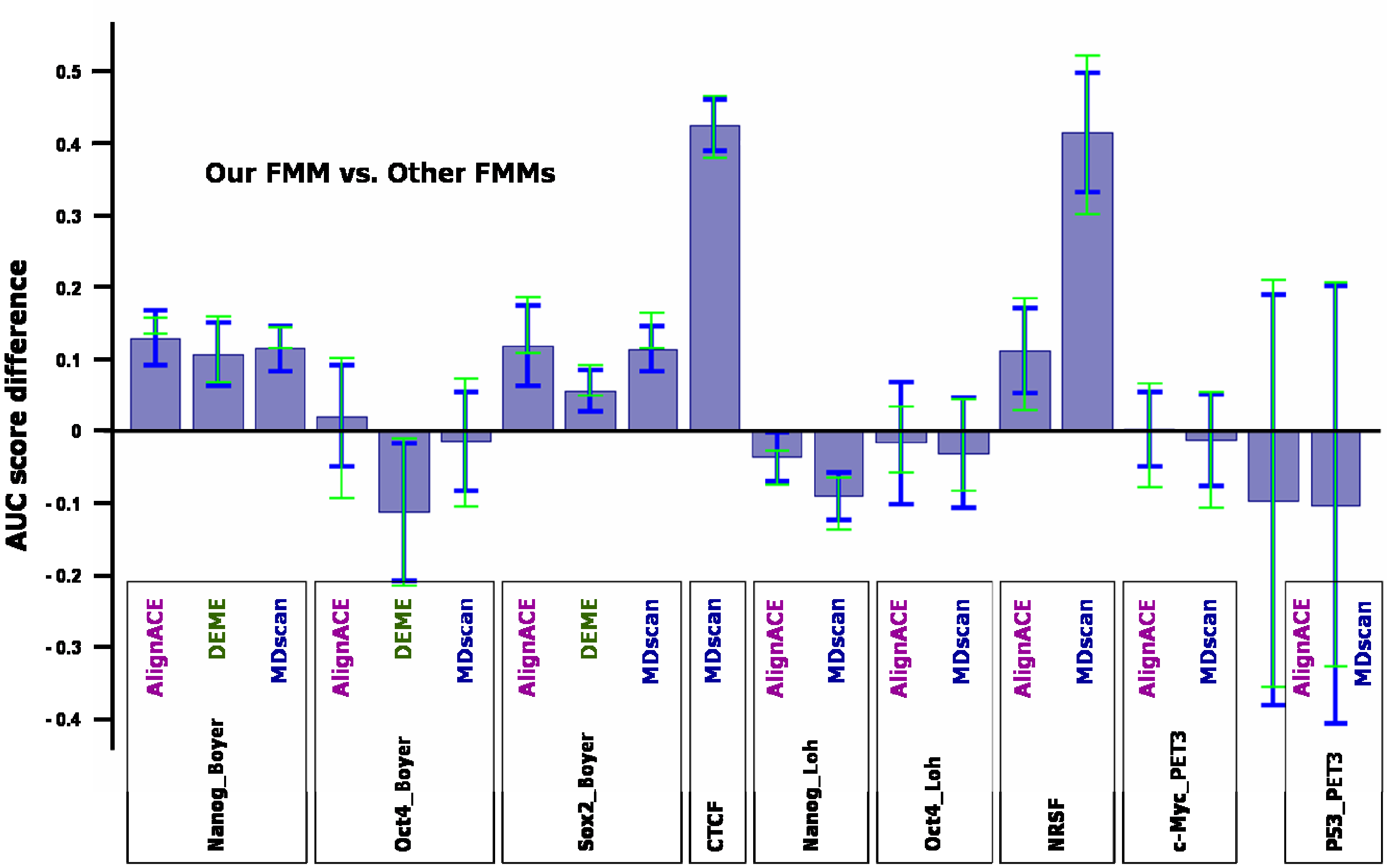

Differences between AUC Scores of our FMMs and the other motif finders FMMs. The means and the standard deviations (calculated over the five CV groups) of the difference between our FMM AUC score and the other tool FMM AUC score. The blue bars and error bars are for AUC scores based on the test data. The light green error bars are for AUC scores based on the train data.

Figure S2

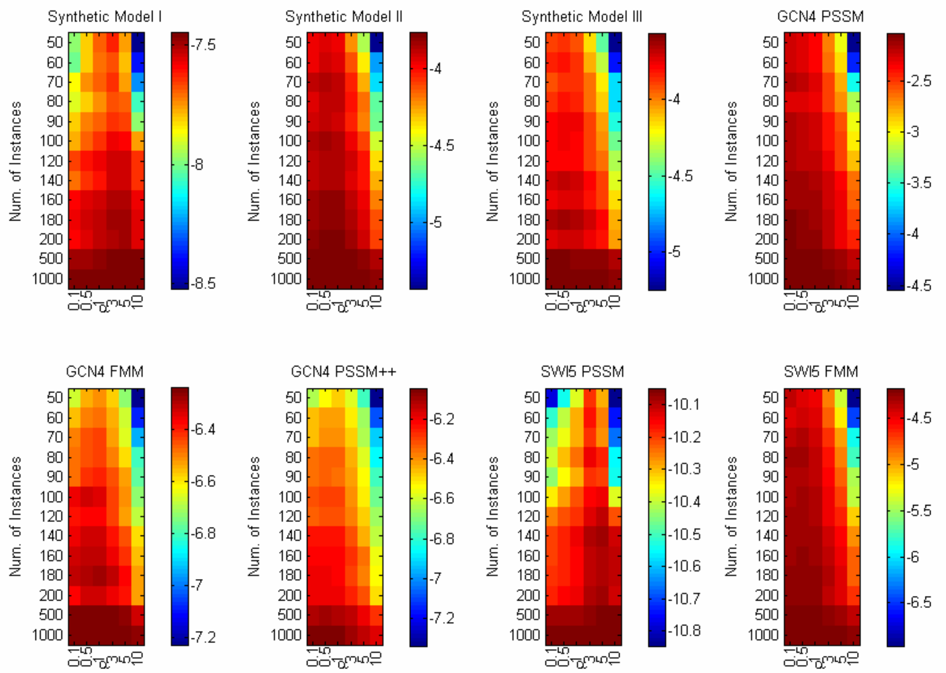

Evaluation Of The L1 Penalty Term Free Parameter On Synthetic Data. FMM model performance in terms of the average test set likelihood on 8 synthetic data sets (sampled from the models in Figure 3) as a function of the number of data instances and the L1 penalty free parameter (α). We observed that the effect of the value of α is, as predicted much stronger on small datasets. Where too small values of α might not prevent overfitting (those resulting in low average test likelihood), too large values might pose to harsh restriction on the learned features. However relativelly small values of α (α=1) have prevented overfitting for PSSM sampled datasets of size 1000. Base on this results we selected the value 1, which gave relativelly good performances on all datasets, for our runs.

De-Novo Motifs. **Figures S9-S23** show a summery of the de-novo found motifs in the examined human and mouse data sets, which are described in **Table 1.**

Figure S9

Data set: c-Myc							
Index	P	PH	N	NH	MHG P-val	PSSM	FMM
1	3996	1487	7992	1072	10^{-189}	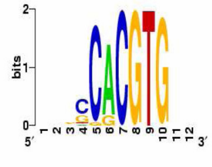	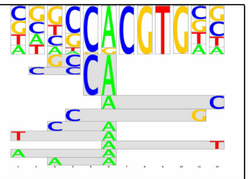
2	3996	711	7992	1014	$3 \cdot 10^{-16}$	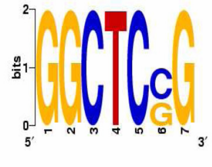	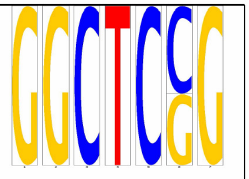
3	3996	547	7992	768	$4 \cdot 10^{-15}$	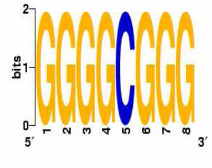	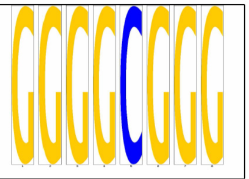
4	3996	338	7992	407	$2 \cdot 10^{-14}$	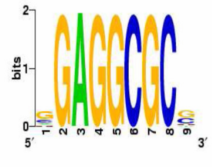	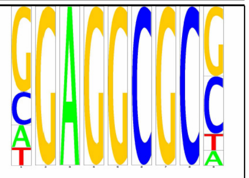
5	3996	322	7992	390	10^{-12}	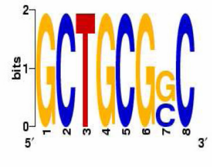	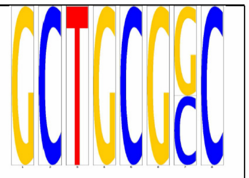

A summary of all de-novo found motifs for the c-Myc dataset. “P”/“N” stand for the number of positive/negative sequences, “PH”/“NH” stand for the number of positive/negative sequences in which there is a KMM hit.

Figure S10

Data set: c-Myc_PET3							
Index	P	PH	N	NH	MHG P-val	PSSM	FMM
1	528	145	1056	69	10^{-30}	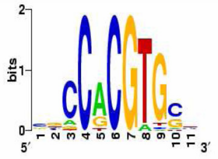	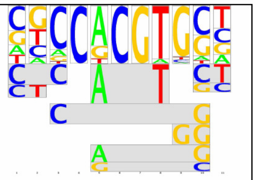
2	528	243	1056	298	10^{-19}	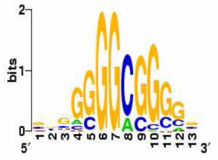	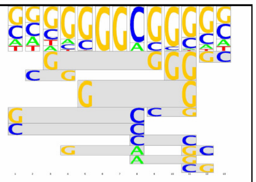
3	528	351	1056	537	$2 \cdot 10^{-13}$	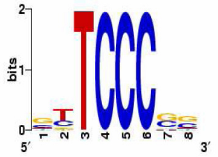	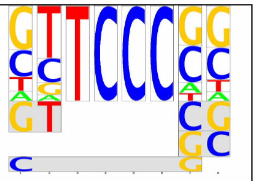
4	528	232	1056	364	$5 \cdot 10^{-13}$	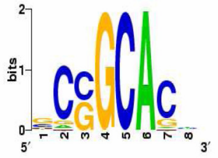	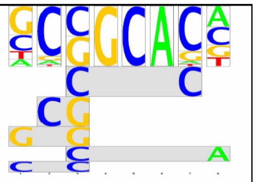
5	528	72	1056	45	$3 \cdot 10^{-11}$	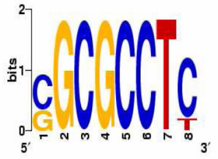	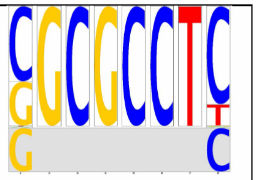

A summary of all de-novo found motifs for the c-Myc_PET3 dataset. “P”/“N” stand for the number of positive/negative sequences, “PH”/“NH” stand for the number of positive/negative sequences in which there is a KMM hit.

Figure S11

Data set: CTCF							
Index	P	PH	N	NH	MHG P-val	PSSM	FMM
1	13721	8235	27442	4750	~ 0	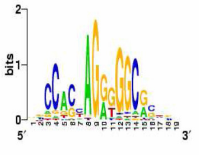	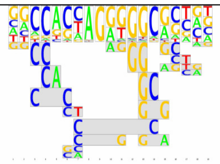
2	13721	4257	27442	4445	10^{-265}	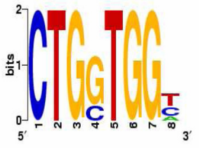	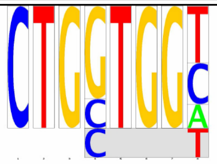
3	13721	10370	27442	16953	10^{-248}	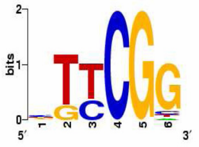	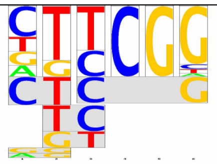
4	13721	7907	27442	11925	-172	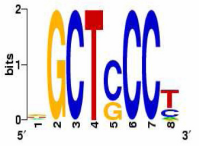	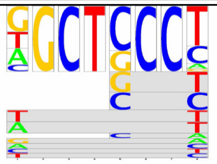
5	13721	3031	27442	3481	10^{-131}	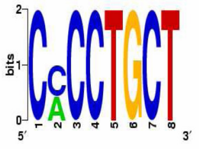	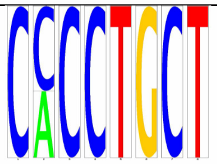

A summary of all de-novo found motifs for the CTCF dataset. “P”/“N” stand for the number of positive/negative sequences, “PH”/“NH” stand for the number of positive/negative sequences in which there is a KMM hit.

Figure S12

Data set: E2F4_Boyer							
Index	P	PH	N	NH	MHG P-val	PSSM	FMM
1	956	891	956	298	10^{-231}	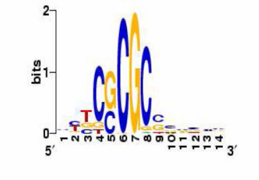	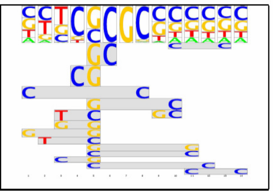
2	956	886	956	617	10^{-81}	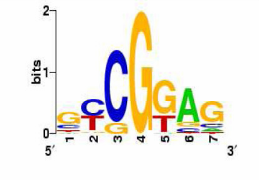	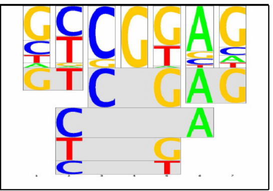
3	956	463	956	175	10^{-47}	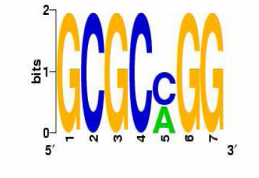	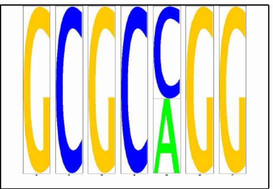
4	956	441	956	217	10^{-39}	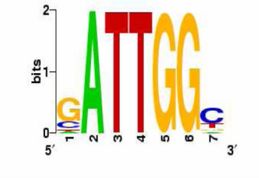	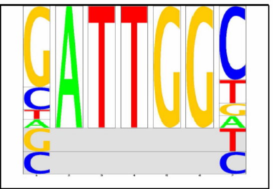
5	956	226	956	69	10^{-24}	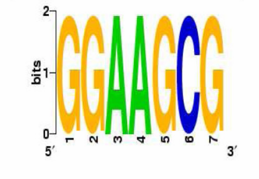	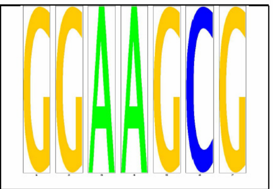

A summary of all de-novo found motifs for the E2F4_Boyer dataset. “P”/“N” stand for the number of positive/negative sequences, “PH”/“NH” stand for the number of positive/negative sequences in which there is a KMM hit.

Figure S13

Data set: NANOG_Boyer							
Index	P	PH	N	NH	MHG P-val	PSSM	FMM
1	1552	164	3104	4	10^{-74}	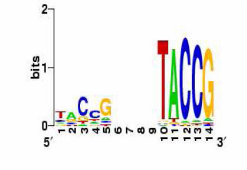	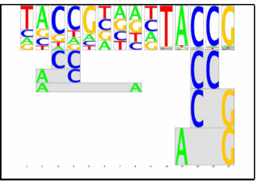
2	1552	1188	3104	1804	10^{-49}	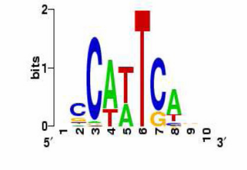	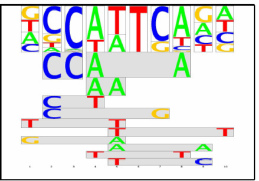
3	1552	551	3104	578	10^{-44}	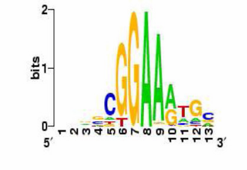	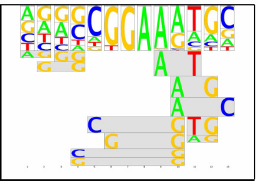
4	1552	739	3104	1039	10^{-25}	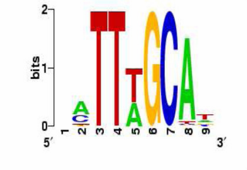	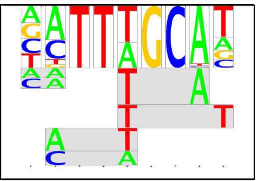
5	1552	501	3104	659	10^{-17}	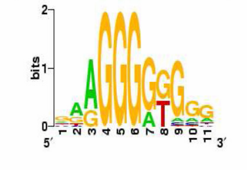	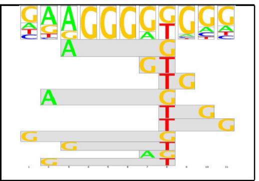

A summary of all de-novo found motifs for the NANOG_Boyer dataset. "P"/"N" stand for the number of positive/negative sequences, "PH"/"NH" stand for the number of positive/negative sequences in which there is a KMM hit.

Figure S14

Data set: NANOG_Loh							
Index	P	PH	N	NH	MHG P-val	PSSM	FMM
1	2823	1250	5646	1245	10^{-109}	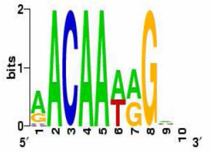	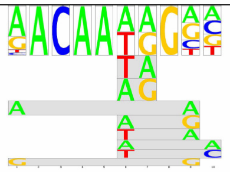
2	2823	441	5646	263	10^{-62}	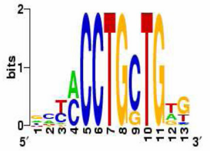	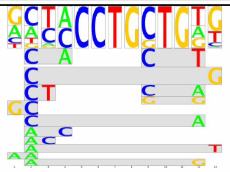
3	2823	606	5646	579	10^{-43}	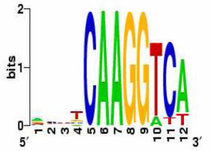	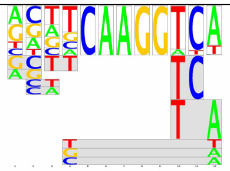
4	2823	88	5646	0	10^{-42}	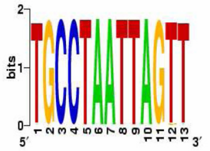	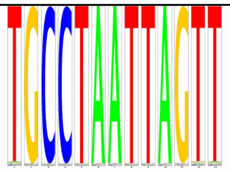
5	2823	1569	5646	2393	10^{-41}	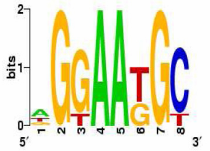	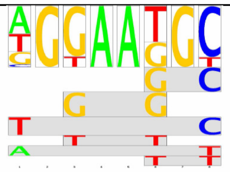

A summary of all de-novo found motifs for the NANOG_Loh dataset. "P"/"N" stand for the number of positive/negative sequences, "PH"/"NH" stand for the number of positive/negative sequences in which there is a KMM hit.

Figure S15

Data set: NRSF							
Index	P	PH	N	NH	MHG P-val	PSSM	FMM
1	1872	1630	3744	358	~ 0	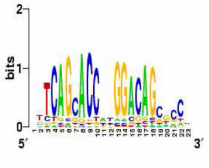	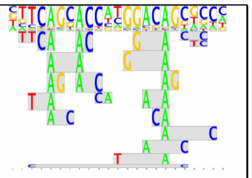
2	1872	245	3744	195	10^{-23}	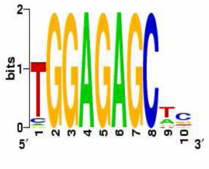	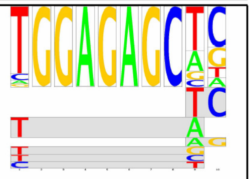
3	1872	63	3744	10	10^{-20}	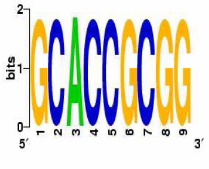	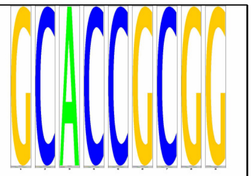
4	1872	138	3744	119	$2 \cdot 10^{-12}$	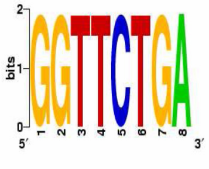	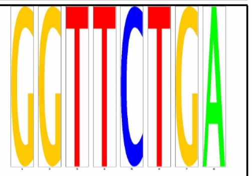
5	1872	198	3744	212	$6 \cdot 10^{-12}$	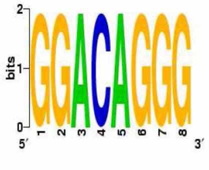	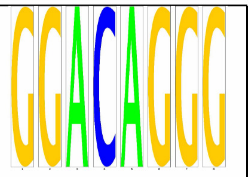

A summary of all de-novo found motifs for the NRSF dataset. "P"/"N" stand for the number of positive/negative sequences, "PH"/"NH" stand for the number of positive/negative sequences in which there is a KMM hit.

Figure S16

Data set: OCT4_Boyer							
Index	P	PH	N	NH	MHG P-val	PSSM	FMM
1	603	349	1206	327	10^{-40}	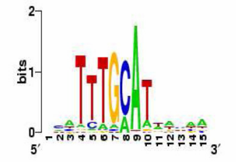	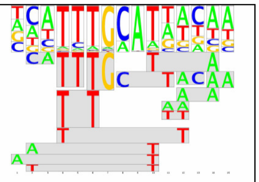
2	603	521	1206	884	$2 \cdot 10^{-14}$	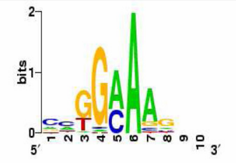	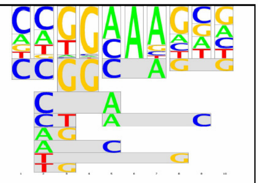
3	603	22	1206	0	$3 \cdot 10^{-11}$	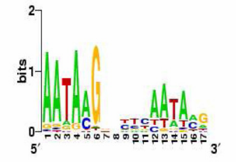	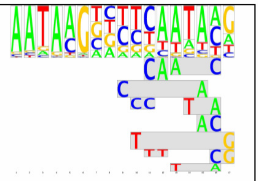
4	603	64	1206	46	$6 \cdot 10^{-9}$	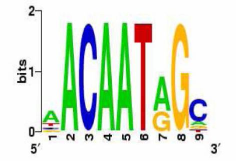	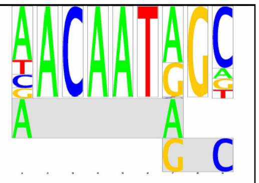
5	603	219	1206	316	$2 \cdot 10^{-6}$	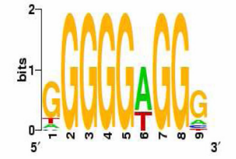	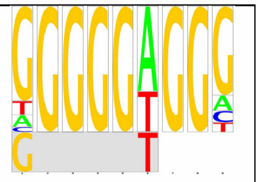

A summary of all de-novo found motifs for the OCT4_Boyer dataset. "P"/"N" stand for the number of positive/negative sequences, "PH"/"NH" stand for the number of positive/negative sequences in which there is a KMM hit.

Figure S17

Data set: OCT4_Loh							
Index	P	PH	N	NH	MHG P-val	PSSM	FMM
1	968	517	1936	474	10^{-58}	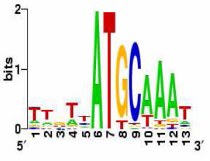	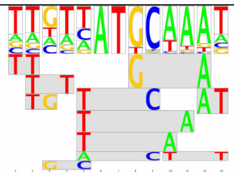
2	968	696	1936	997	10^{-28}	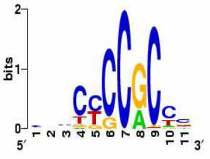	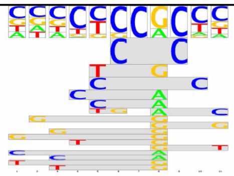
3	968	437	1936	536	10^{-23}	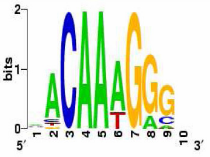	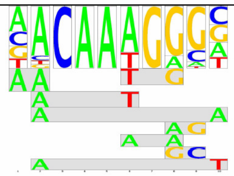
4	968	714	1936	1242	$4 \cdot 10^{-14}$	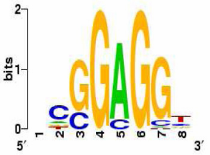	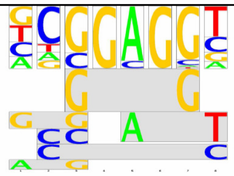
5	968	291	1936	383	$6 \cdot 10^{-11}$	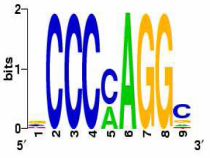	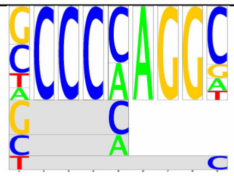

A summary of all de-novo found motifs for the OCT4_Loh dataset. “P”/“N” stand for the number of positive/negative sequences, “PH”/“NH” stand for the number of positive/negative sequences in which there is a KMM hit.

Figure S18

Data set: P53							
Index	P	PH	N	NH	MHG P-val	PSSM	FMM
1	503	433	1006	163	10^{-164}	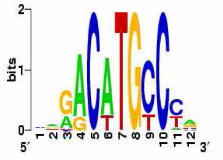	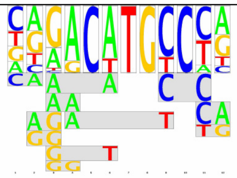
2	503	194	1006	198	10^{-20}	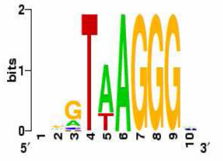	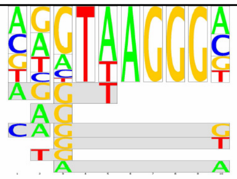
3	503	115	1006	88	10^{-18}	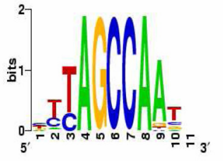	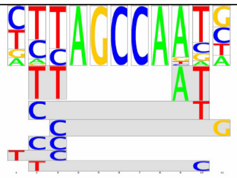
4	503	130	1006	134	$3 \cdot 10^{-14}$	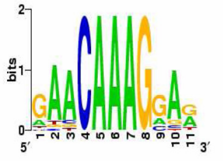	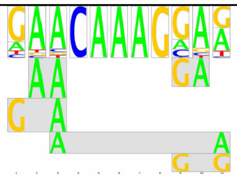
5	503	95	1006	94	$5 \cdot 10^{-9}$	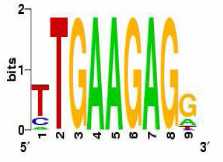	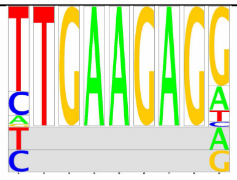

A summary of all de-novo found motifs for the P53 dataset. "P"/"N" stand for the number of positive/negative sequences, "PH"/"NH" stand for the number of positive/negative sequences in which there is a KMM hit.

Figure S19

Data set: P53_PET3							
Index	P	PH	N	NH	MHG P-val	PSSM	FMM
1	300	228	600	66	10^{-87}	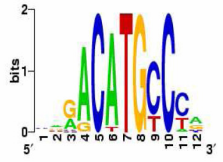	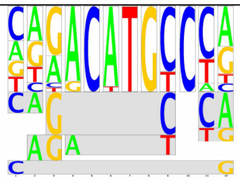
2	300	86	600	2	10^{-41}	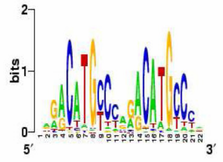	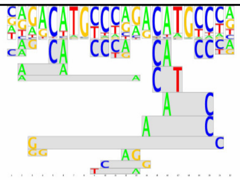
3	300	64	600	51	$2 \cdot 10^{-10}$	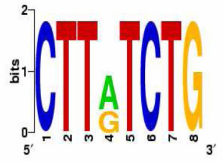	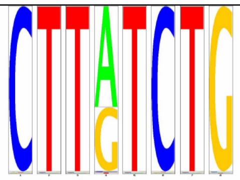
4	300	99	600	114	10^{-10}	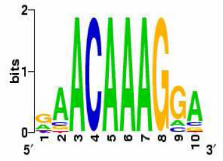	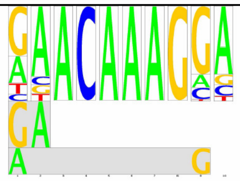
5	300	66	600	49	$2 \cdot 10^{-9}$	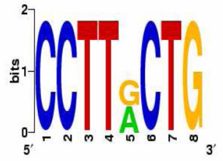	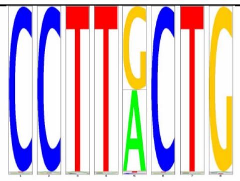

A summary of all de-novo found motifs for the P53_PET3 dataset. “P”/“N” stand for the number of positive/negative sequences, “PH”/“NH” stand for the number of positive/negative sequences in which there is a KMM hit.

Figure S20

Data set: PRC2_SUZ12							
Index	P	PH	N	NH	MHG P-val	PSSM	FMM
1	2905	2315	2905	971	10^{-317}	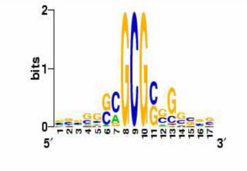	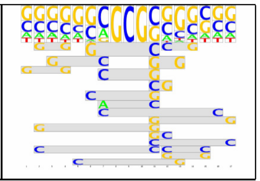
2	2905	2162	2905	1428	10^{-120}	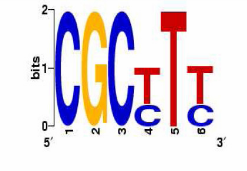	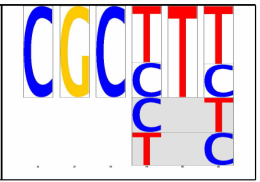
3	2905	991	2905	497	10^{-53}	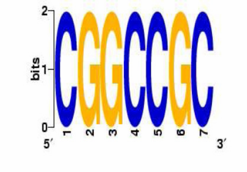	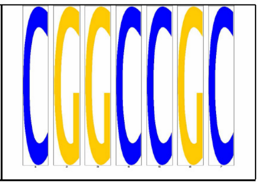
4	2905	428	2905	154	10^{-34}	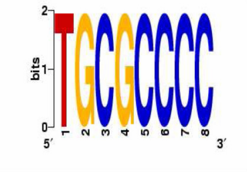	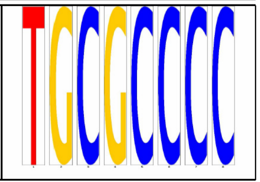

A summary of all de-novo found motifs for the PRC2_SUZ12 dataset. “P”/“N” stand for the number of positive/negative sequences, “PH”/“NH” stand for the number of positive/negative sequences in which there is a KMM hit.

Figure S21

Data set: SOX2_Boyer							
Index	P	PH	N	NH	MHG P-val	PSSM	FMM
1	1165	686	2330	678	10^{-72}	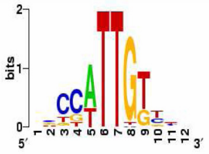	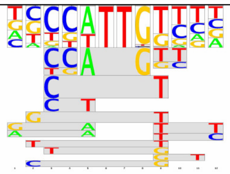
2	1165	600	2330	831	10^{-25}	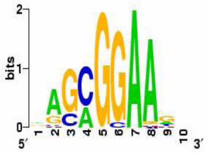	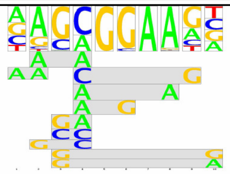
3	1165	354	2330	390	10^{-22}	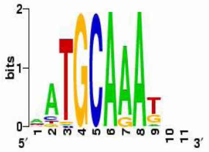	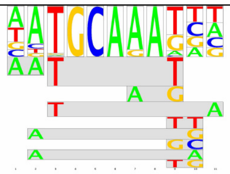
4	1165	739	2330	1189	10^{-14}	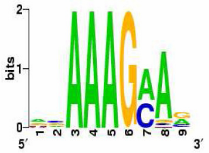	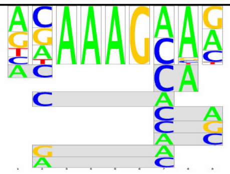
5	1165	149	2330	130	$2 \cdot 10^{-13}$	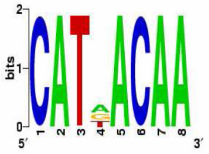	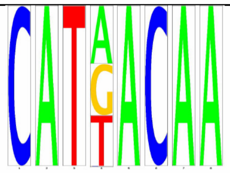

A summary of all de-novo found motifs for the SOX2_Boyer dataset. "P"/"N" stand for the number of positive/negative sequences, "PH"/"NH" stand for the number of positive/negative sequences in which there is a KMM hit.

Figure S22

Data set: STAT1_IFNg							
Index	P	PH	N	NH	MHG P-val	PSSM	FMM
1	15000	4392	15000	1895	10^{-303}	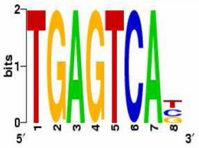	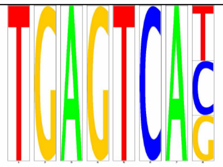
2	15000	4347	15000	2055	10^{-289}	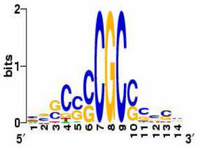	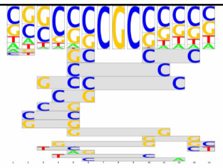
3	15000	2265	15000	601	10^{-252}	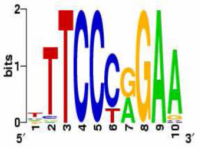	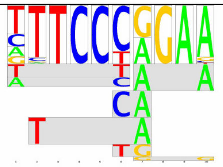
4	15000	3425	15000	1913	10^{-123}	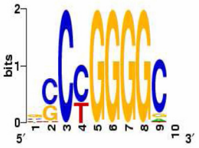	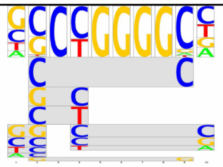
5	15000	2074	15000	1179	10^{-64}	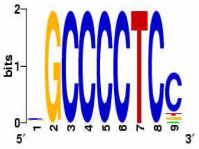	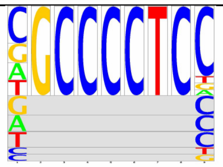
6	15000	868	15000	378	10^{-47}	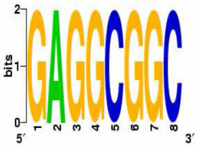	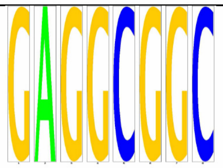

A summary of all de-novo found motifs for the STAT1_IFNg dataset. “P”/“N” stand for the number of positive/negative sequences, “PH”/“NH” stand for the number of positive/negative sequences in which there is a KMM hit.

Figure S23

Data set: STAT1_Unstimulated							
Index	P	PH	N	NH	MHG P-val	PSSM	FMM
1	11004	4581	22008	3745	~ 0	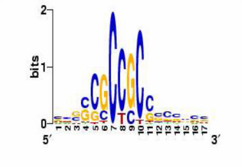	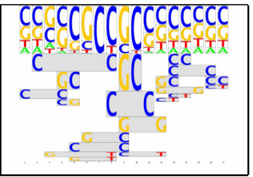
2	11004	1390	22008	691	10^{-253}	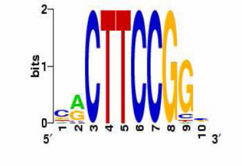	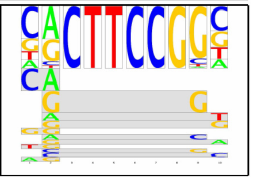
3	11004	2074	22008	1747	10^{-196}	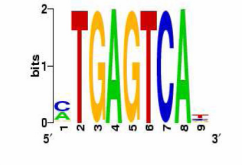	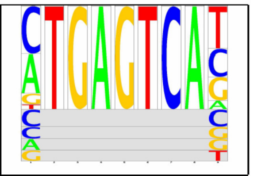
4	11004	933	22008	689	10^{-94}	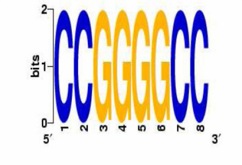	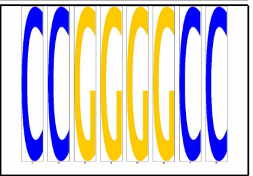

A summary of all de-novo found motifs for the STAT1_Unstimulated dataset. "P"/"N" stand for the number of positive/negative sequences, "PH"/"NH" stand for the number of positive/negative sequences in which there is a KMM hit

References

1. Hughes JD, Estep PW, Tavazoie S, Church GM (2000) Computational identification of cis-regulatory elements associated with groups of functionally related genes in Saccharomyces cerevisiae. J Mol Biol 296(5): 1205-1214.
2. Liu XS, Brutlag DL, Liu JS (2002) An algorithm for finding protein-DNA binding sites with applications to chromatin-immunoprecipitation microarray experiments. Nature Biotechnology 20: 835-839.
3. Redhead E, Bailey TL (2007) Discriminative motif discovery in DNA and protein sequences using the DEME algorithm. BMC Bioinformatics 8: 385-403.
